# Attention deficit in primary-school-age children with attention deficit hyperactivity disorder measured with the attention network test: a systematic review and meta-analysis

**DOI:** 10.3389/fnins.2023.1246490

**Published:** 2023-12-07

**Authors:** Łucja Bieleninik, Gabriela Gradys, Angel M. Dzhambov, Tamara Walczak-Kozłowska, Kornelia Lipowska, Ariadna Łada-Maśko, Katarzyna Sitnik-Warchulska, Paulina Anikiej-Wiczenbach, Michał Harciarek, Małgorzata Lipowska

**Affiliations:** ^1^Institute of Psychology, Faculty of Social Sciences, University of Gdańsk, Gdańsk, Poland; ^2^GAMUT-The Grieg Academy Music Therapy Research Centre, NORCE Norwegian Research Centre AS, Bergen, Norway; ^3^Institute of Pedagogy and Languages, University of Applied Sciences in Elbla̧g, Elbla̧g, Poland; ^4^Institute of Applied Psychology, Faculty of Management and Social Communication, Jagiellonian University, Kraków, Poland; ^5^Environmental Health Division, Research Institute at Medical University of Plovdiv, Medical University of Plovdiv, Plovdiv, Bulgaria; ^6^Research Group “Health and Quality of Life in a Green and Sustainable Environment,” Strategic Research and Innovation Program for the Development of MU–Plovdiv, Medical University of Plovdiv, Plovdiv, Bulgaria; ^7^Department of Hygiene, Faculty of Public Health, Medical University of Plovdiv, Plovdiv, Bulgaria; ^8^Institute of Highway Engineering and Transport Planning, Graz University of Technology, Graz, Austria; ^9^Department of Psychology, Faculty of Social and Behavioural Sciences, University of Amsterdam, Amsterdam, Netherlands

**Keywords:** ADHD, attention deficit hyperactivity disorder, attention network task, ANT, a systematic review and meta-analysis

## Abstract

**Objective:**

To review and meta-analyze patterns of attention deficit in primary-school-age children with ADHD measured with the neuropsychological attention network test (ANT).

**Methods:**

Six electronic databases were searched to 5.05.2022. Selection criteria included prospective cohort and intervention studies; ANT used; primary-school-age; diagnosis of ADHD/at high risk.

**Results:**

Seven studies met inclusion criteria (*N* = 3,826). Compared with controls, children with ADHD had higher scores for Reaction Time (Hedges’ *g* = 0.433; 95% CI: 0.135–0.731), Reaction Time Variability (Hedges’ *g* = 0.334; 95% CI: 0.012–0.657), and Alerting Network (Hedges’ *g* = 0.235; 95% CI: 0.021–0.449) while children at high risk had higher Alerting Network scores (Hedges’ *g* = 0.176; 95% CI: 0.003–0.349) and Correctness scores (Hedges’ *g* = 1.956; 95% CI: 0.020–3.892).

**Conclusions:**

Children with ADHD and at risk of ADHD had different ANT results from children without ADHD only for the alerting network. There were no significant differences for executive and orienting outcomes. Children at risk of ADHD also made more errors (commission and omission) measured with the ANT compared with children without ADHD. Reaction time was longer and reaction time variability higher in children with ADHD than in children without ADHD, and in children at risk of ADHD compared with children without ADHD.

**Preregistration:**

A protocol has been registered with the International Prospective Register of Systematic Reviews (PROSPERO) database (registration number: CRD42021249768).

## 1 Introduction

### 1.1 Background

Attention deficit hyperactivity disorder (ADHD) is a neurodevelopmental disorder that can take one or both of two forms: attention deficit disorder (ADD) and/or hyperactivity and impulsiveness (American Psychiatric Association, 2013). ADHD is one of the most common neurodevelopmental disorders in school-aged children, mostly between 6 and 9 years of age; however, symptoms can also manifest in adulthood (Roberts et al., 2015). According to Thomas et al. (2015), ADHD affects approx. 6.7–7.8% of the population; as such, there are typically 1–3 individuals with ADHD symptoms in a class of 25–30 children (DuPaul et al., 2014). ADHD is a significant risk factor for poor academic achievement (Daley and Birchwood, 2010): in comparison with students without disabilities, students with ADHD more frequently repeat a grade, are referred for special education services, are suspended from school, and drop out of school (Siqueira and Gurge-Giannetti, 2011). It seems that efficient attentional abilities are a prerequisite for good school outcomes. Moreover, ADHD affects other areas of a person’s functioning (both in childhood and adolescence). Children with ADHD may experience relationship difficulties: they are more often excluded from groups and make fewer friends compared to their typically-developing peers. Additionally, as pointed out by Lee et al. (2021), the friendships they do make are of poorer quality–they tend to last for a shorter period of time. According to Harpin (2005), children with ADHD, in comparison to their typically-developing peers, more often present difficulties in relations with their parents and siblings. Furthermore, parents of children with ADHD more often suffer from depression and experience less satisfaction with their role than do parents of children without this disorder (Harpin, 2005). These problems that children with ADHD experience in their lives can lead to lower self-esteem as well as difficulties regulating behavior and dealing with emotions. In adult life, they are more often dismissed from employment, have interpersonal issues with their colleagues, and are at greater risk of drug and substance abuse as well as other mental diseases (Harpin, 2005).

The attentional deficits have significant negative impact on the everyday lives of those diagnosed with ADHD. So far, the neurological basis of ADHD has not been clearly identified (Weyandt and Gudmundsdottir, 2015). According to the American Psychiatric Association (2013), ADHD may result from deficits in various attentional capabilities. Brandt (2019) indicates that there is a lack of neuropsychological methods in specialist diagnostics that would allow reliable distinctions between “endogenous” attentional deficits in ADHD (resulting from abnormal brain development) and “exogenous” attentional deficits, which can appear during the lifespan as a result of exposure to toxins, unhealthy habits such as suboptimal nutrition (Brandt, 2019), spending a lot of time in front of screens, poor sleep, other neurodevelopmental and psychiatric disorders (Krakowski et al., 2020; Rutter and Arnett, 2021), challenging life experiences (Barnow et al., 2007; Bouchard and Saint-Aubin, 2014), or acute brain damages (Roberts et al., 2019). Moreover, it is a challenge to distinguish between attentional deficits associated directly with the efficiency of attentional networks observed at the neurological level (using clinical judgment based on clinical trials) with those observed at behavioral level (using self-reported instruments such as difficulties in staying alert during lecture or following the instructions, trouble getting organized, always “on the go,” talkative). There are few clinical instruments that are used in the diagnosis of attentional deficits in children at behavioral level (e.g., the Continuous Performance Test, Child Behavior Checklist), and there are no measurements available for the diagnosis of attentional deficits at the neurological level (specifically, the efficiency of attentional networks). The attention network test (ANT) is a neuropsychological tool that can be useful in the measurement of this capacity; however, efforts are needed to investigate the performance of this test more thoroughly (Vázquez-Marrufo et al., 2019).

### 1.2 Attention network test

The ANT is a computer-based task predicated on the neuropsychological theory of the attention system (Posner and Petersen, 1990). In this approach, the attention system consists of three independent and integrated attentional networks: the alerting network–the ability to maintain increased sensitivity to new incoming stimuli; the orienting network–the ability to select stimuli and focus attention on the stimulus of interest; and the conflict or executive network–the ability to control a behavioral response in response to a stimulus that enables two alternative responses. As indicated by Fan et al. (2002), the ANT measures the efficiency of the three attentional networks by gathering information on the correctness and the reaction time of a participant’s responses to presented stimuli. In this task, participants react to the target arrows by pressing arrow keys on a keyboard (pointing in the same direction as the arrow on the screen). In some trials, the arrows are preceded by various visual cues and/or warning tones. There are three types of flankers (neutral, compliant, and inconsistent) that are preceded by one of four types of clues (none, double, center, and spatial). Researchers can make adjustments to the rules, such as the time of presentation of the fixation, signal, target, and final fixation slides. There are several versions of the ANT that have been developed over the past two decades: ANT-C (Rueda et al., 2004), LANT (Greene et al., 2008), ANT-R (Fan et al., 2009), ANT-I (Callejas et al., 2004), and ANTI-V (Roca et al., 2011). Although these versions measure in general the same outcome, the differences from the original version concern the terminology used, and some aspects and characteristics of the task. In the child version of the ANT (Child-ANT or ANT-C), the target presented is a fish that, in some trials, is surrounded by four distractor fish (Rueda et al., 2004). Gradys et al. (2022) shows a diagram based on the original ANT (Fan et al., 2002) and another previous research (Rueda et al., 2004; Vázquez-Marrufo et al., 2019).

### 1.3 Rationale

To the best of our knowledge, few studies have been conducted on the measurement of attentional deficits in children with ADHD using the Attentional Network Test. Deficits in attentional capabilities in children with ADHD do not necessarily apply to the entire attention system. They may refer to specific sub-components of that system. Careful investigation of deficits in the three attentional networks allow the precise identification of the areas of attention that function sub-optimally in children with ADHD. This knowledge may facilitate the use of the most effective treatments. Currently in the literature there exists a single narrative review (Vázquez-Marrufo et al., 2019) and a single meta-analysis (Arora et al., 2020) on this subject; however, the results of these papers were inconsistent and neither of them followed the Preferred Reporting Items for Systematic Review and Meta-analysis (PRISMA) statement (Liu et al., 2019). Moreover, Mullane and Klein (2008) conducted systematic review with a meta-analysis in children with and without ADHD, but they focused in general on flanker and Simon task performance (they did not include ‘ANT’ or ‘Attention Network Test’ or ‘Attention Network Task’ in search terms)–probably because the authors were focused mainly on interference control effects in this cohort. Among the 12 studies included by Mullane and Klein (2008) only two used ANT (in child and adult versions). The outcomes included in meta-analysis were computed using Reaction Time variability and percentage of correct answers for congruent and incongruent trails, but did not include Reaction Time and percentage of correct answers for trails with warning cues (probably because in other than ANT type of flanker task no warning cues were provided). Of note, this systematic review also did not follow the PRISMA statement and authors highlighted in the limitation section that further studies with a more rigorous meta-analytic review should be conducted. Thus, it remains unclear whether there are any differences in ANT performance between children with and without ADHD. It also needs to be elucidated how the differences manifest and the direction of changes.

The aim of this systematic review was to identify a single distinctive pattern of ANT performance in children with ADHD, including the efficiency of attentional networks, reaction time, and numbers of all types of errors. Such an approach would allow the comparison of ANT results obtained in future experimental studies with the pattern observed in ADHD respondents in our systematic review; with this knowledge it would be possible to determine whether such patterns are the result of the heterogeneity of the study group. The results of this review and metanalysis will make it possible to use the ANT in further research and perhaps serve as a basis for standardization of the test, which could make the ANT useful for broad diagnoses of attention.

### 1.4 Research questions

This meta-analysis seeks to examine patterns of attention deficit using ANT among children with ADHD or at risk of ADHD compared to controls. The primary research question was as follow: Compared to children without ADHD, do primary-school-age children diagnosed with ADHD or at risk of ADHD demonstrate differences in the efficiency of functioning of the three attention networks measured with the ANT?

This meta-analysis addressed the following secondary research questions:

• Compared to children without ADHD, do primary-school-age children diagnosed with ADHD or at risk of ADHD demonstrate differences in Correctness scores (numbers of Commission and Omission Errors) measured with the ANT?

1. Compared to children without ADHD, do primary-school-age children diagnosed with ADHD or at risk of ADHD demonstrate differences in the number of mistakes made in particular clue or flanker tasks measured with the ANT?
2. Compared to children without ADHD, do primary-school-age children diagnosed with ADHD or at risk of ADHD demonstrate differences in reaction times, in particular clue or flanker tasks measured with the ANT?
3. Compared to children without ADHD, do primary-school-age children diagnosed with ADHD or at risk of ADHD demonstrate differences in results of the ANT depending on the version of ANT used, e.g., ANT-C (Rueda et al., 2004), LANT (Greene et al., 2008), ANT-R (Fan et al., 2009), ANT-I (Callejas et al., 2004), ANTI-V (Roca et al., 2011).

## 2 Methods

The inclusion criteria, search strategy, and analysis methods were specified in advance, published in a protocol (Gradys et al., 2022), and registered with the International Prospective Register of Systematic Reviews (PROSPERO) database (registration number: CRD42021249768). We undertook a comprehensive literature search following the Preferred Reporting Items for Systematic Review and Meta-analysis for Protocols (PRISMA-P) guidelines (Moher et al., 2009; Page et al., 2021b). Amendments of the protocol can be found in Supplementary Table 1. This report has been prepared in accordance with the PRISMA statement (Page et al., 2021a,b). The PRISMA checklist is shown in Supplementary Table 2.

### 2.1 Eligibility criteria

Studies were selected according to pre-specified eligibility criteria following the Population, Intervention, Comparison, Outcomes, and Study model (PICOS model; see Table 1).

**TABLE 1:** Eligibility criteria.

Criterion	Description
Participants	Individuals of primary school age [age range between 5 and 13 years following The International Standard Classification of Education 2011 (Schneider, 2013)], without restriction to gender, nationality, who had an ADHD diagnosis or were considered to be at high risk of ADHD. ADHD should have been diagnosed based on DSM-5 (ADHD diagnostic code: 314) (American Psychiatric Association, 2013) either earlier versions of the DSM or the International Classification of Diseases (ICD, ADHD diagnostic code according to the ICD-11: 6A05) (World Health Organization, 2019) by a specialist (psychiatrist, clinical psychologist, or any other qualified medical staff). The risk of ADHD could be confirmed by ADHD symptoms following questionnaires such as CONNERS-3, Structured Diagnostic Interview Questionnaire for ADHD – or foreign equivalents. Comorbidities such as anxiety disorder, conduct disorder, learning disorder, and oppositional defiant disorder were allowed because of the nature of common coexistence with ADHD at the primary school age. However, children with other atypical concomitants or concurrent disorders, such as eating disorders, depressive or bipolar disorders, obsessive-compulsive disorders, or factitious disorders, were excluded because of the potential impact on the ADHD core symptoms and ANT performance.
Intervention/Exposure	Any type of intervention for which the effectiveness was measured using the ANT.
Comparator/Control	Children without ADHD (without ADHD nor at risk of ADHD) at primary school age, both sexes, without restriction to nationality
Outcomes	Performance of any version of the ANT, e.g., ANT-C (Rueda et al., 2004), LANT (Greene et al., 2008), ANT-R (Fan et al., 2009), ANT-I (Callejas et al., 2004), ANTI-V (Roca et al., 2011). *Primary outcomes:* Mean and standard deviation or median and range (or standardized effect measures such as Cohen’s d) of the executive, alerting, and orienting attention network, measured by the ANT. *Secondary outcomes:* Mean and standard deviation or median and range and intra-individual variability (or standardized effect measures such as Cohen’s d) of general reaction time achieved in the ANT. A number of omissions (missing answers) and a number of commissions (wrong answers) errors or if there will be a lack of that data, general correctness rate (percent of the correct answer) reached in ANT.
Study Type	Prospective cohort studies and prospective studies of intervention effects with a control group (both randomized and non-randomized controlled)
Settings/Location	Without any restrictions on the area of the conducted research. The individuals could have been recruited from both primary schools or health care facilities (such as primary care settings, therapeutic settings, or diagnostic settings)
Country	Any
Date	From the earliest available
Language	Any

### 2.2 Information source and search strategy

We searched both electronic databases (PubMed, PsychInfo, Web of Science, EMBASE, DARE, and the Cochrane Library) and the reference lists of included review articles to identify any additional studies. The literature search was conducted up to the 8th of May 2021 and updated on the 5th of May 2022 following the same search strategy. The search was not restricted to any language, sample size, or year of publication. We excluded editorials, letters, case studies, case series, and conference abstracts. The initial search strategy was piloted on PubMed. The following search strings were used: (“attention deficit disorder with hyperactivity” OR ADHD) AND (“Attentional Network Test” OR “Attentional Networks Test” OR “Attentional Network Task” OR “Attentional Networks task” OR “Attention Network Test” OR “Attention Networks Test” OR “Attention Network Task” OR “Attention Networks task”). More details can be found in the protocol (Gradys et al., 2022).

### 2.3 Selection process

Study selection was carried out by one reviewer (GG) who searched electronic databases and manually searched the reference list of the included review articles. All potentially relevant records were extracted to EndNote reference management software (Eapen, 2006) and duplicates were identified and deleted. At the next stage, titles and abstracts were checked for their eligibility for inclusion by two review authors (GG, TW-K) independently. Then, the full texts of the studies found with the aforementioned eligibility criteria were read by two review authors (GG, TW-K) independently. The review authors provided the reason(s) for rejection. Eligibility criteria for each study were assessed in order of importance, starting with participants, followed by the outcome, intervention/exposure, comparator/control, and study design. In this strategy, the first ‘no’ response constitutes the primary reason for excluding a study, and the remaining criteria are not assessed. Any discrepancies at the screening stage and eligibility phases were resolved by discussion with another reviewer (ŁB). During the study selection, the review authors were not blinded to the journal titles, study authors, or their institutions. To resolve issues with eligibility, the review authors sought additional information from the study’s corresponding authors where necessary.

### 2.4 Data collection process

Data were extracted from the included studies based on a specifically designed and pre-piloted data extraction form by at least two review authors, working in pairs (KS-W, KL, PA-W, AŁ-M). Data extractors were trained in code entry and procedures. The data extraction stage was performed by area experts from the field of clinical psychology who are familiar with the ANT. Any discrepancies were resolved by consultation with a third reviewer (GG).

#### 2.4.1 Duplicate publications

Multiple reports based on the same study were merged based on matching the following study characteristics: author names, location and setting, number of participants and baseline data, and duration of the study. In the case of multiple reports based on the same project, we extracted data from each report separately and combined information across multiple data collection forms afterward.

#### 2.4.2 Requesting missing data

Corresponding authors were requested to provide any missing data by e-mail. The strategy is described in the study protocol (Gradys et al., 2022). We attempted to contact the authors of 10 of the 18 included studies for clarification or to request missing data. We successfully obtained the requested data only in 3 cases. If we did not receive any feedback, we considered the data to be unobtainable.

### 2.5 Data items

The following data were extracted.

1. Publication details: author; year of publication; country in which the study was conducted.
2. The number of participants per group (clinical–ADHD/risk of ADHD; control–non-ADHD).
3. Characteristics of the clinical population: age; sex; ADHD group type (ADHD/risk of ADHD); diagnosis provider; diagnosis method(s); diagnosis criteria; comorbidities; pharmacotherapy (yes/no); pharmacotherapy used during ANT assessment (yes/no; withdrawal time before testing).
4. Characteristics of the control population: age, sex.
5. Study design: prospective cohort study/intervention study.
6. ANT results: means and standard deviations or medians and ranges (or standardized effect measures such as Cohen’s d) of the scores for Executive, Alerting, and Orienting attention networks; mean and standard deviation or median and range, as well as intra-individual variability, of general Reaction Time; numbers of Omission Errors (missing answers) and Commission Errors (wrong answers) or, in the absence of this data, general Correctness Rate (percent correct answers). We extracted baseline data in observational studies with repeated measurements or intervention studies with several time points. We gathered data on the version of the ANT used, how the training for the ANT was performed, how the instructions were presented, the person conducting the test and their interventions with the child during the test, as well as any other descriptive data about ANT performance and the administration of the test.

### 2.6 Synthesis methods

Data analysis included three ANT factors:

(1) Correctness (hereafter referred to as Errors)–measured by the number of Omission Errors (missing answers) and number of Commission Errors (wrong answers) or, in the absence of this data, the general Correctness Rate (percent correct answers) achieved by the children.
(2) Reaction Time–mean and standard deviation or median and range and intra-individual variability of general experiment reaction time.
(3) Attention Network–measured by the difference in mean or median reaction times between:
  (a) Double clue vs. no clue or tone vs. no tone (Alerting Network);
  (b) Valid clue vs. invalid clue (Orienting Network);
  (c) Congruent vs. incongruent trial type (Executive Network).

These three factors (Correctness, Reaction Time, and Attention Network) were plotted in order to compare data between the ADHD clinical group and the control group, as well as between the group at risk of ADHD and the control group, in order to detect potential specific differences in children with ADHD and at risk of ADHD, to thereby establish a characteristic pattern of ANT results for children with ADHD answering the study questions.

We have provided a narrative synthesis of the included studies in a comparative table structured around the type of participants, study design, and ANT performance. Quantitative data were combined when means and standard deviations were available or could be derived from the available data.

### 2.7 Meta-analysis

We planned to use the fixed-effects estimator in the absence of materially important heterogeneity; otherwise, we would use the restricted maximum likelihood random estimator. We combined only studies for which the effect estimates could be converted to a common metric–specifically, group means and standard deviations into Hedges’ *g*. Some studies (Booth et al., 2007; Kooistra et al., 2011; Antonini et al., 2016) reported means and SDs separately for ADHD-I and ADHD-C subgroups, so they were combined for the meta-analysis using methods provided in the Cochrane Handbook (Higgins et al., 2022). Kooistra et al. (2011) reported median test scores, but since we lacked sufficient information on the distribution of these scores, we could not convert them to means (Wan et al., 2014). Instead, we used them as means and conducted sensitivity analyses by excluding these estimates to check the robustness of the meta-analysis.

Statistical heterogeneity in the models was suggested by a significant Cochran’s *Q* at the *p* < 0.1 level and quantified by the *I*^2^ statistic as follows: *mild* – < 30%, *moderate* – 30–50%, or *high* – > 50% (Higgins and Thompson, 2002). We also inspected the direction of individual study effect sizes and the overlap of their confidence intervals. The quantitative synthesis was carried out using Stata v. 17 (College Station, TX: StataCorp LP) and MetaXL v. 5.3 (EpiGear International Pty Ltd., Sunrise Beach, Queensland, Australia).

### 2.8 Sensitivity analyses

For meta-analysis models with more than 2 studies, we conducted a leave-one-out meta-analysis to determine whether excluding studies one at a time would materially change the pooled effect. This way we could identify studies that had a strong influence on the results.

Given growing concerns about the appropriateness of the random effects model and its potential to yield overly liberal findings, we also re-ran the meta-analysis using the inverse-variance heterogeneity model; this model is built under the fixed effect model assumption with a quasi-likelihood based variance structure to retain correct coverage probability and yield more conservative pooled estimates regardless of heterogeneity (Doi et al., 2015, 2017).

### 2.9 Study quality assessment

The Newcastle-Ottawa Scale (NOS) for cohort studies was used to assess the study quality by two review authors (ML, MH) independently. Any discrepancies were resolved by experts in the fields of clinical trial methodology (ŁB) or psychology (TW-K). The NOS assesses the following three items: selection – representativeness of the exposed cohort, selection of the non-exposed cohort, ascertainment of exposure, demonstration that the outcome of interest was not present at the start of the study; the comparability of cohorts on the basis of the design or analysis; and the outcome – assessment of outcome, follow-up, adequacy of follow up of cohorts (Wells et al., 2014). Each study can be awarded a maximum of one star for each numbered item within the selection and outcome categories. A maximum of two stars can be given for comparability, giving a maximum of 9 stars. To evaluate the quality of the studies, we applied the “good,” “fair,” and “poor” thresholds for converting NOS assessments to the Agency for Healthcare Research and Quality (AHRQ) guidelines (2012). Studies rated 3 or 4 stars in the selection domain, 1 or 2 stars in the comparability domain, and 2 or 3 stars in the outcome/exposure domain were considered to represent *good* quality. Studies rated 2 stars in the selection domain, 1 or 2 stars in the comparability domain, and 2 or 3 stars in the outcome/exposure domain were considered to represent *fair* quality. Papers evaluated as being 0 or 1 stars in the selection domain, 0 stars in the comparability domain, and 0 or 1 stars in the outcome/exposure domain were considered to be *poor* quality.

### 2.10 Overall quality of evidence

We followed the Grading of Recommendations, Assessment, Development, and Evaluations (GRADE) approach (Schünemann et al., 2013; McKenzie and Brennan, 2022) to grade the quality of evidence for each outcome. The quality of evidence was assessed by two review authors (ML, KS-W) independently for each outcome based on the following domains: (1) high risk of bias across the studies; (2) indirectness of evidence; (3) high heterogeneity or inconsistency of results across studies; and (4) imprecision of results. Any discrepancies were resolved by a third reviewer (ŁB). The quality of evidence was judged based on the extent to which we could be certain that the pooled effect estimate is close to the true effect as follows: *high evidence* (the authors had a lot of confidence that the true effect is similar to the estimated effect), *moderate evidence* (the authors believed that the true effect is probably close to the estimated effect), *low evidence* (the true effect might be markedly different from the estimated effect), or *very low evidence* (the true effect is probably markedly different from the estimated effect). The grading process across domains and reasons for up or downgrading can be found in Supplementary Table 3. As established in the systematic review protocol (Gradys et al., 2022), observational studies were assumed to start at “moderate” quality, which could be upgraded or downgraded in subsequent steps.

## 3 Results

### 3.1 Study selection

**FIGURE 1:**
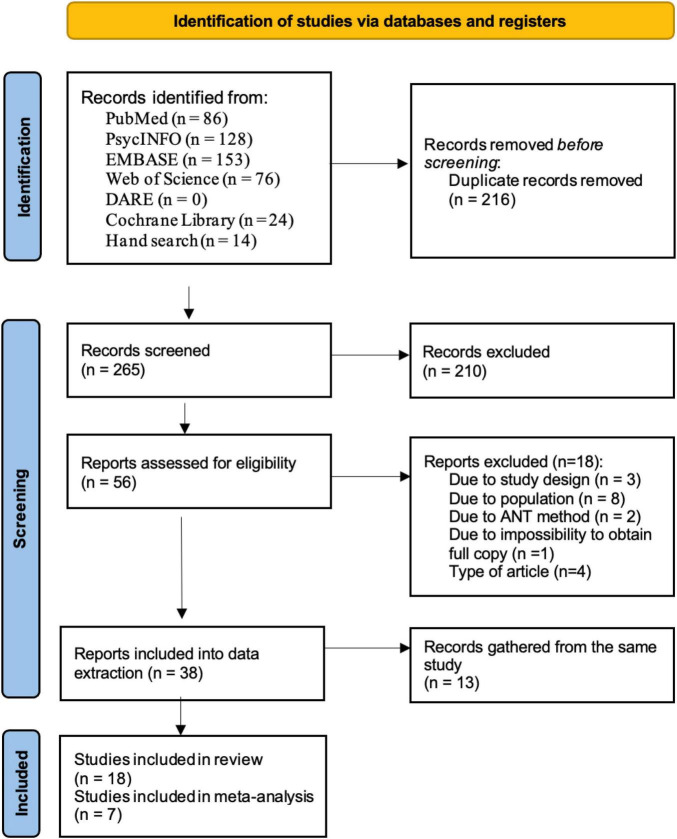
PRISMA flow diagram of the study selection process.

Up to 15th of May 2021, we identified a total of 422 records in our database search (PubMed – 82; PsycINFO – 121; EMBASE – 132; Web of Science – 70; DARE – 0; the Cochrane Library – 23) and 14 studies from searching their reference lists. An update and final search were performed on the 8th of May 2022 and identified 39 additional studies (PubMed – 4; PsycINFO – 7; EMBASE – 21; Web of Science – 6; DARE – 0; Cochrane Library – 1; manual search – 0). The results of both rounds of database searches are presented in Figure 1.

After excluding 216 duplicates, 265 articles were screened and 56 full texts were assessed for eligibility. At this stage, 18 records did not meet the pre-specified eligibility criteria and were excluded (the list of excluded studies along with reasons for exclusion is reported in Supplementary Table 4). A total of 38 reports were advanced to data extraction. Thirteen publications based on data from the same project were merged together (see Supplementary Table 5 for the list of duplicate studies and reasons for merging), leaving 18 studies included in the systematic review (Supplementary Table 6) and seven appropriate for quantitative synthesis/meta-analysis (Booth et al., 2007; Adólfsdóttir et al., 2008; Kooistra et al., 2011; Forns et al., 2014; Hansen et al., 2014; Mogg et al., 2015; Antonini et al., 2016).

### 3.2 Study characteristics

The final sample for the systematic review included 18 prospective cohort studies (Supplementary Table 6). We did not find studies of intervention effects with a control group, even though we planned to include such studies. Table 2 shows the characteristics of the studies. Of the 18 studies included, three were conducted in Norway (Adólfsdóttir et al., 2008; Forssman et al., 2009; Hansen et al., 2014), three in Canada (Kooistra et al., 2011; Mullane et al., 2011; Waldon et al., 2018), two in the USA (Booth et al., 2007; Antonini et al., 2016), two in Brazil (Mogg et al., 2015; Abramov et al., 2019), two in Spain (Forns et al., 2014; Julvez et al., 2020), two in Germany (Konrad et al., 2010; Kratz et al., 2011), and one each from Ireland (Johnson et al., 2008), Poland (Racicka-Pawlukiewicz et al., 2021), India (Gupta et al., 2011), and China (Chen et al., 2022).

**TABLE 2:** Characteristics of the 18 studies included in a systematic review.

Refe-rences	Country	Study design	*N*	Sex, N male	Mean (range) age in years	ADHD vs. risk of AHDH	ADHD types	Comorbi-dities	Comorbi-dities – addi-tional comments	Diag-nosis provider	Diag-nosis method	Diag-nosis Criteria	Pharma-cotherapy (% of N; with-drawal time before testing)	ANT type used	Study findings (only signifi-cant)
Abramov et al., 2019	Brazil	CS	39 Clinical: 19 Control: 20	100 Clinical: 100 Control: 100	11.41 (10.46–12.6)	ADHD	–	without	–	Psychiatrist	Interview	DSM IV-TR	- 30 days before	ANT-C	Worse Executive Network, Worse Alerting Network, Less Accuracy, More Variety of Reaction time
Adólfsdóttir et al., 2008	Norway	CS	102 Clinical: 45 Control: 57	60 Clinical: 71 Control: 50	10.1 (9.0–11.1)	ADHD	–	without	–	Accessor, Psychiatrist, Psychologist	DAWBA, Interview, Questioners, SNAP-IV	DSM-IV	20% Drugs was taken during study	ANT-C	Less accuracy More Variation of Reaction Time
Antonini et al., 2016	USA	CS	147 Clinical: 102 Control: 45	72 Clinical: 73 Control: 71	8.59 (6.95–9.63)	Risk of ADHD	Risk of ADHD-C, Risk of ADHD-I	Anxiety, CD, Emotional, ODD	In combine group: ODD – 48% Anxiety – 38% Mood – 2% All – 70% In Inattention group: ODD – 63% CD – 8% Anxiety – 33% Mood – 2% All – 88% In Control group: 5% Anxiety	Accessor, Teacher	DISC-P, Questioners, Vanderbilt ADHD Teacher Rating Scale	DSM IV-TR	–	ANT-C	Worse Executive Network, Less Accuracy, More Variety of Reaction time
Booth et al., 2007	USA	CS	66 Clinical: 42 Control: 24	68 Clinical: 77 Control: 50	9.5 (7.6–11.4)	ADHD	ADHD-C, ADHD-I	LD, ODD	In ADHD-I: LD 4% ODD 19% in ADHD-C: LD 12.5% ODD 56% in Control group: ODD 4% – in Control group	Accessor, Psychiatrist, Psychologist, Teacher	Interview, Questioners	DSM-IV	58% of ADHD-I and 88% of ADHD-C 18 H before	ANT-C	Lower Accuracy, Slower Reaction Time, Worst Alerting Network
Chen et al., 2022	China	CS	75 Clinical: 39 Control: 36	61 Clinical: 74 Control: 42	11.11 (8.7–10.1)	Risk of ADHD	–	without	–	Accessor, Teacher	Conners, SNAP-IV	DSM-IV	–	ANT-I	Worse Alerting Network, Worse Executive Network, Slower Reaction Time, Less accuracy
Forns et al., 2014	Spain	CS	2582 Clinical: 271 Control: 2311	61 Clinical: 74 Control: 48	8.64 (7.67–9.41)	Risk of ADHD	–	–	–	Teacher	Questioners	DSM-IV	–	ANT-C	Worse Conflict Network, Less Accuracy
Forssman et al., 2009	Norway	CS	221 Clinical: -Control: –	64.7 Clinical: - Control: –	9.87 (8.88–10.86)	Risk of ADHD	–	–	–	Accessor	Interview, Questioners, SNAP-IV	DSM-IV	–	ANT-C	–
Gupta et al., 2011	India	CS	240 Clinical: 120 Control: 120	–	MD (6–9)	ADHD	–	ODD	20% of ADHD group	Accessor, Psychiatrist	Conners, Interview	DSM-IV	–	ANT-C	Data combined from different tests – no information only of ANT
Hansen et al., 2014	Norway	CS	73 Clinical: 38 Control: 35	67 Clinical: 76 Control: 57	10.25 (8.3–13.1)	ADHD	–	–	–	Accessor, Psychologist	ASEBA TRF, Interview, K-SAD-PL	DSM-IV	Never used by study subjects	ANT-C	Slower Reaction Time, Worse Alerting Network
Johnson et al., 2008	Ireland	CS	–	–	-	ADHD	–	CD, ODD	52% ODD/CD of ADHD	Accessor, Psychiatrist	Conners, Interview, Questioners	DSM-IV	- 24H before	ANT-C	Worse Alerting Network, Worse Executive Network, Slower Reaction Time, Less Accuracy
Julvez et al., 2020	Spain	CS	1674 Clinical: 329 Control: 1345	–	8	Risk of ADHD	–	–	–	Accessor	Conners	DSM IV-TR	–	ANT-C	–
Konrad et al., 2010	Germany	CS	302 Clinical: 181 Control: 121	70 Clinical: 72 Control: 68	10.85	ADHD	ADHD-C, ADHD-H, ADHD-I	OD, CD, Emotional disorders, Tic, Anxiety	CD – 4% ODD – 30% Mood – 3% Anxiety – 9% Tic – 3% Overall – 49%	Accessor, Psychiatrist	CBCL, FBB-HKS, Interview	DSM-IV	Drugs taken during test	ANT-C	Worse Executive Network
Kooistra et al., 2011	Canada	CS	85 Clinical: 47 Control: 38	64 Clinical: 80 Control: 53	9.4 (7.15–11.0)	ADHD	ADHD-C, ADHD-I	LD	51% of all ADHD had LD	Accessor, Paediatrician, Psychiatrist	Conners, DICandA-IV, DISC-P, Interview, SAC	DSM-IV	91% of all ADHD, 24-h before	ANT-C	Worse Executive Networks, Less Accuracy
Kratz et al., 2011	Germany	CS	44 Clinical: 25 Control: 19	77.5 Clinical: 76 Control: 79	9.95 (8.7–11.2)	ADHD	ADHD-C, ADHD-I	Emotional Disorders, LD, ODD	In Both groups: Dyslexia 16% OOD 8% Emot.Dis 8%	Psychiatrist, Psychologist	FBB-HKS, Interview, Questioners	DSM-IV	Never used by study subjects	ANT-C	Slower Reaction Time, More Variety of Reaction Time
Mogg et al., 2015	Brazil	CS	771 Clinical: 45 Control: 726	53.5 Clinical: 53 Control: 54	9.7 (7.6–11.9)	ADHD	ADHD and Control	CD, ODD	Study subject was divided by 2 groups – ADHD without comorbidities and ADHD with CD/ODD. Subjects with another Comorbidities was excluded from study	Accessor, Psychiatrist	CBCL, DAWBA, Interview	DSM-IV	Never used by study subjects	ANT-C	Worse Executive Network, Worse Orienting Network, Less Accurate, More Variety of Reaction Time
Mullane et al., 2011	Canada	CS	90 Clinical: 45 Control: 45	68 Clinical: 67 Control: 69	9.39 (6.75–12.42)	ADHD	ADHD and Contro, ADHD-C, ADHD-I	Anxiety, LD, ODD	ADHD-I: 8/20 LD ADHD-C: 4/25 comorbidities (LD, ODD, Anxiety) 8% ODD, 4% Anxiety, 4% LD, 4% LD + Anxiety	Psychologist	Conners, Interview, Questioners	DSM IV-TR	24	ANT-I	Worse Alerting Network, Worse Executive Network, Slower Reaction Time, Less Accurate
Racicka-Pawlukiewicz et al., 2021	Poland	CS	–	–	–	ADHD	ADHD and Control	CD, LD, ODD, Tic	71% of all ADHD with some comorbidities	Psychiatrist	Interview	ICD	- Taken during test	ANT	Worse Executive Network, Slower Reaction Time, Less Accurate
Waldon et al., 2018	Canada	CS	50 Clinical: 25 Control: 25	78 Clinical: 80 Control: 76	9.7 (7.4–10.9)	ADHD	ADHD and Control	–	–	Accessor, Psychologist	Conners, Interview	DSM-IV	Never used by study subjects	ANT-I	Worse Alerting Network, Worse Executive Network

Study IDs marked in gray were included in the main meta-analysis. ANT, Attention Network Test; ANT-C, Attention Network Test-Continuous Performance; ANT-I, Attention Network Test-Interactions; ASEBA, Achenbach System of Empirically Based Assessment, a set of questionnaires used to assess behavioral and emotional problems in children and adults; CD, conduct disorder; Conners, Conners’ Rating Scales, a series of standardized instruments used to assess symptoms of ADHD and related disorders; SCS, cohort study; DAWBA, Development and WellBeing Assessment, an interview and questionnaire used to diagnose psychiatric disorders in children and adolescents; DISC-P, Diagnostic Interview Schedule for Children-Parent Version, an interview used to diagnose psychiatric disorders in children; DSM – IV, Diagnostic and Statistical Manual of Mental Disorders, 4th edition; DSM-IV-TR, Diagnostic and Statistical Manual of Mental Disorders, 4th edition, text revision; FBB-HKS, Fragebogen zur Beurteilung von hyperkinetischen Störungen. Questionnaire for the assessment of ADHD symptoms; ICD, International Classification of Diseases; KSAD-PL, Kiddie Schedule for Affective Disorders and Schizophrenia-Present and Lifetime Version, an interview used to diagnose psychiatric disorders in children and adolescents; LD, learning disability; MD, Mood Disorder; ODD, oppositional defiant disorder; SAC, Social Adjustment Scale, a self-report questionnaire used to assess an individual’s social adjustment; SNAP-IV, Swanson, Nolan, and Pelham Rating Scale, a behavioral rating scale used to assess symptoms of ADHD.

#### 3.2.1 Participant characteristics

The pooled sample for meta-analysis included 3,826 participants: 3,236 controls, 217 children diagnosed with ADHD by a specialist (psychiatrist, clinical psychologist, or any other qualified medical practitioner) and 373 children at risk of ADHD recognized by a teacher or parent. The sample sizes ranged from 66 to 2,582 participants with a median of 85. The mean age was 9.45 years (range 6.95–13.1). In the ADHD group, 72% of the participants were boys, but in the control group, the proportion of both sexes was equal. In one of the studies (Adólfsdóttir et al., 2008), the children from the ADHD group did not have any comorbidities; four of the studies (Booth et al., 2007) reported common ADHD-related comorbidities – two (Booth et al., 2007; Kooistra et al., 2011) mentioned learning disorders (LD), three (Booth et al., 2007; Mogg et al., 2015; Antonini et al., 2016) mentioned oppositional defiant disorder (ODD) and two (Booth et al., 2007; Antonini et al., 2016) mentioned conduct disorder (CD). Two studies did not report any information about comorbidities (Forns et al., 2014; Hansen et al., 2014). In summary, 29% of children with ADHD included in this investigation had LD or ODD and at least 21% of children at risk of ADHD had CD or ODD.

#### 3.2.2 ANT characteristics

Most studies included in the systematic review used the ANT version for children – only four studies used a different version: ANT-I (Mullane et al., 2011; Waldon et al., 2018; Chen et al., 2022) or ANT (Racicka-Pawlukiewicz et al., 2021). All seven studies included in the meta-analysis used ANT-C, the version of ANT for children (Rueda et al., 2004), but differed in terms of drug use while taking the ANT. One of these studies (Adólfsdóttir et al., 2008) allowed children with ADHD to take medication during the assessment; two of them (Booth et al., 2007; Kooistra et al., 2011) made sure that children did not take any medication for at least 18 or 24 h before the test; two of the studies (Hansen et al., 2014; Mogg et al., 2015) did not take any medication during the ANT procedure, and the last two of the studies (Antonini et al., 2016) did not mention anything about drug use during the ANT.

### 3.3 Results

#### 3.3.1 Executive, alerting, and orienting networks

**FIGURE 2:**
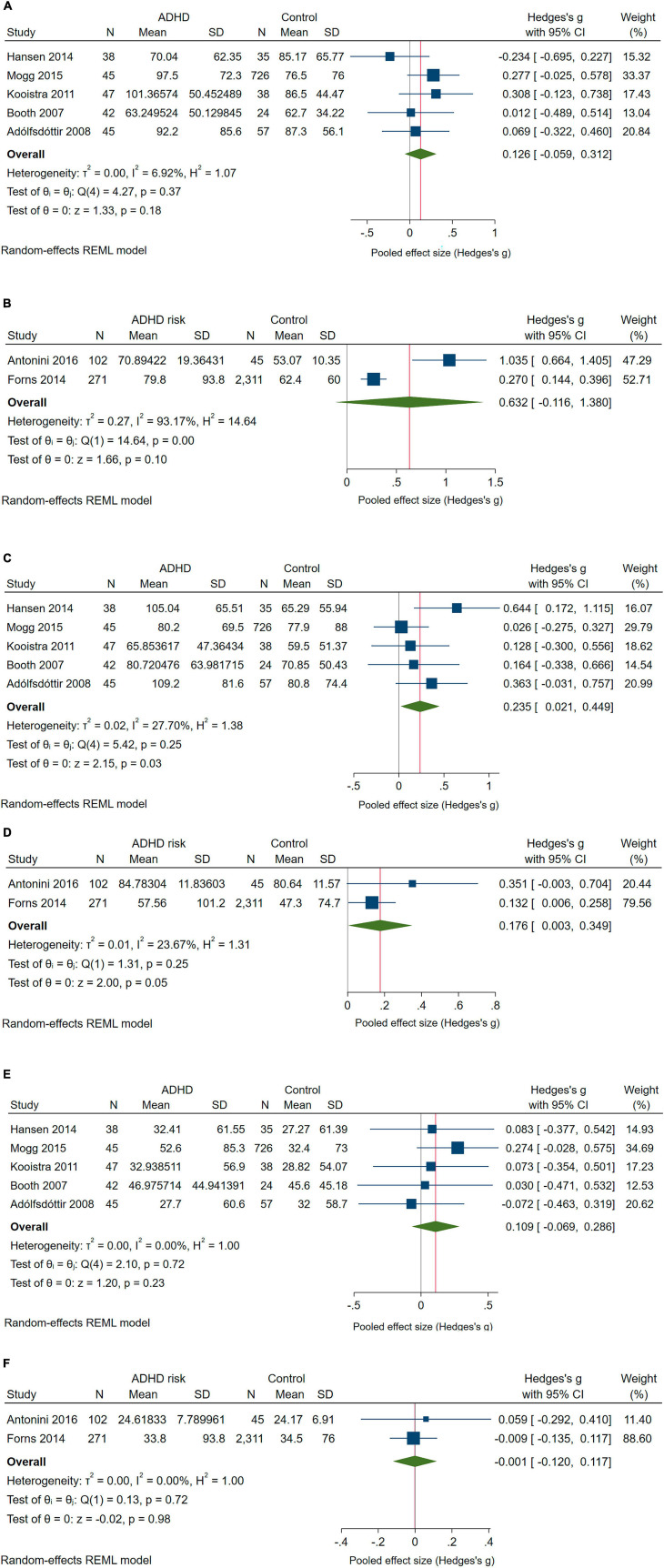
Forest plot with the meta-analysis of the mean differences in Network scores of ANT: **(A)** Executive Network scores of children with ADHD compared with controls; **(B)** Executive Network scores of children at risk of ADHD compared with controls; **(C)** Alerting Network scores of children with ADHD compared with controls. **(D)** Alerting Network scores of children at risk of ADHD compared with controls; **(E)** Orienting Network scores of children with ADHD compared with controls. **(F)** Orienting Network scores of children at risk of ADHD compared with controls.

Five of the seven included studies (Booth et al., 2007; Adólfsdóttir et al., 2008; Kooistra et al., 2011; Hansen et al., 2014; Mogg et al., 2015) reported comparisons of Executive, Alerting, and Orienting Network scores between children with ADHD and controls (Figures 2A–F). Two other studies reported comparisons with children at risk of ADHD (Forns et al., 2014; Antonini et al., 2016) were included in the forest plots with the meta-analysis of the mean differences in network scores of ANT for executive, alerting, and orienting networks.

The pooled difference in Executive Network scores between children with ADHD and controls was not significant in the main meta-analysis (Figure 2A). However, in leave-one-out meta-analysis, excluding the paper by Hansen et al. (2014) – the only study showing lower scores in children with ADHD – increased the effect to *g* = 0.193 (95% CI: 0.001–0.386). Under the alternative IVhet estimator, the pooled effect was similar to the effect under the random effects model (*g* = 0.130; 95% CI: −0.055 to 0.315). Comparing children at risk of ADHD to controls did not yield a significant difference (Figure 2B), and, given the high heterogeneity, the IVhet model produced an even smaller pooled effect compared with the main RE meta-analysis (*g* = 0.349; 95% CI: −0.581 to 1.280).

Significant pooled effects were found for Alerting Network scores, which were higher in both children with ADHD (Figure 2C) and children at risk of ADHD (Figure 2D) compared with their counterparts. However, excluding Booth et al. (2007), Adólfsdóttir et al. (2008), or Hansen et al. (2014) reduced the pooled effect to non-significance, reaching *g* = 0.148 (95% CI: −0.045 to 0.341) if Hansen et al. (2014) was excluded. However, under the IVhet model, the pooled effect held (*g* = 0.219; 95% CI: 0.006–0.432). The effect on Alerting Network scores for children at risk of ADHD vs. controls was not significant with the IVhet estimator (*g* = 0.156; 95% CI: −0.021 to 0.334).

For the Orienting Network, test scores did not differ significantly between children with ADHD or children at risk of ADHD and controls (Figures 2E, F). This did not change if studies were excluded one-at-a-time or if the IVhet estimator was used instead.

#### 3.3.2 Correctness

**FIGURE 3:**
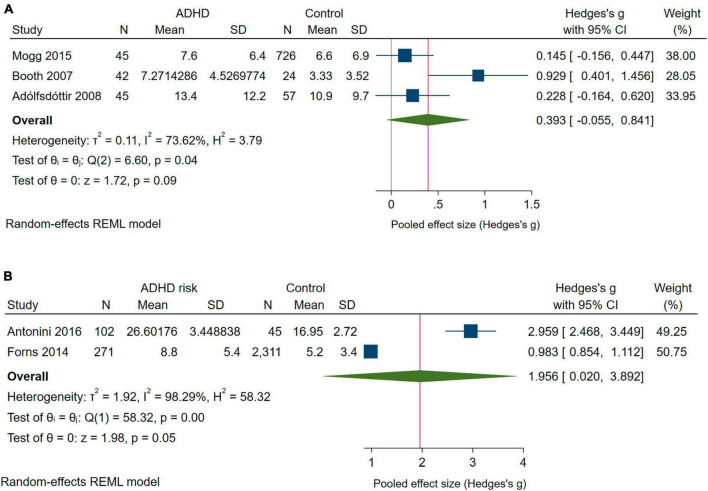
Forest plot with the meta-analysis of the mean differences in Error scores **(A)** of children with ADHD compared with controls **(B)** of children at risk of ADHD compared with controls.

Three studies (Booth et al., 2007; Adólfsdóttir et al., 2008; Mogg et al., 2015) provided estimates of Error scores for children with ADHD and two studies (Forns et al., 2014; Antonini et al., 2016) did so for children at risk of ADHD (Figures 3A, B). For children with ADHD, the effect was borderline significant and largely driven by the results of Booth et al. (2007). The IVhet model did not change this. However, significantly higher Error scores were observed in children at risk of ADHD vs. controls, with Hedges’ *g* indicative of a large effect (Figure 3B); this effect became non-significant under the IVhet model (*g* = 1.110; 95% CI: −1.439 to 3.659).

#### 3.3.3 Reaction time and reaction time variability

**FIGURE 4:**
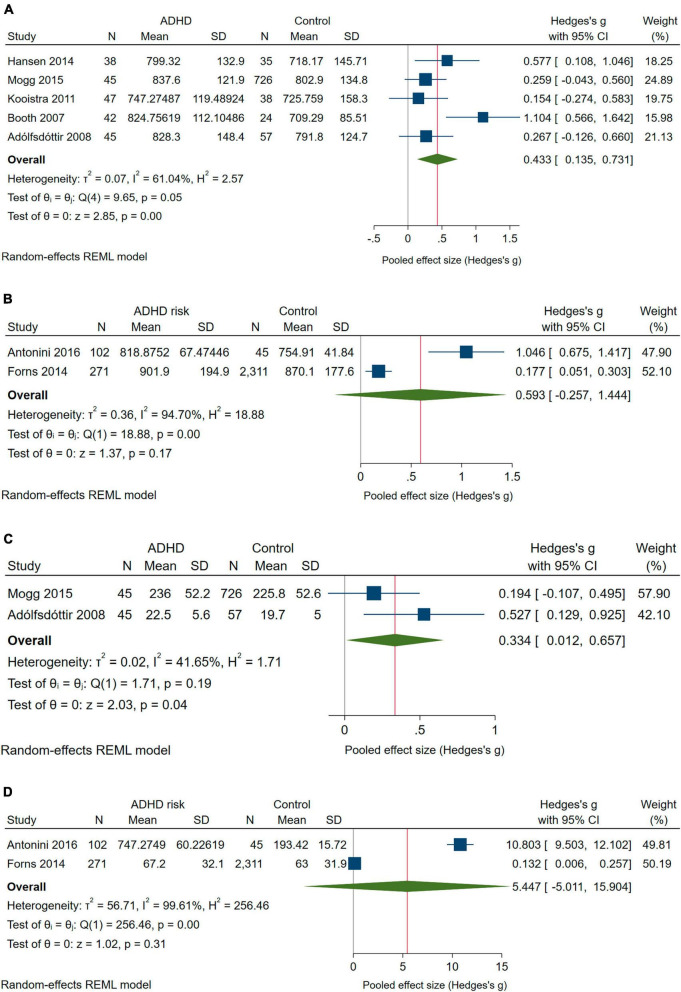
Forest plot with the meta-analysis of the mean differences in Reaction time and Reaction time variability: **(A)** Reaction time of children with ADHD compared with controls. **(B)** Reaction time of children at risk of ADHD compared with controls. **(C)** Reaction Time Variability scores of children with ADHD compared with controls. **(D)** Reaction Time Variability scores of children at risk of ADHD compared with controls.

Five studies compared Reaction Time between children with ADHD and controls (Booth et al., 2007; Adólfsdóttir et al., 2008; Kooistra et al., 2011; Hansen et al., 2014; Mogg et al., 2015); for the other three outcomes, only two studies provided useful estimates (Forns et al., 2014; Antonini et al., 2016). Figures 4A–D show the pooled results for Reaction Time and Reaction Time Variability. Reaction Time was longer and Reaction Time Variability higher in children with ADHD than in controls (Figures 4A, B). The pooled effect for Reaction Time in children with ADHD was driven by Booth et al. (2007), the exclusion of which reduced it to *g* = 0.292 (95% CI: 0.102–0.483), and the IVhet model also produced a smaller effect size (*g* = 0.383; 95% CI: 0.086–0.681) but remained significant in both sensitivity analyses. As for the effect on Reaction Time Variability, it became borderline significant with the IVhet model (*g* = 0.315; 95% CI: −0.009 to 0.640).

For children at risk of ADHD, the pooled effects went in the same direction but were not significant (Figures 4C, D). The IVhet model reduced the pooled effect for Reaction Time to *g* = 0.267 (95% CI: −0.797 to 1.331) and even more so the pooled effect for Reaction Time Variability (*g* = 0.231; 95% CI: −14.394 to 14.855).

#### 3.3.4 ANT version

We were not able to get an answer to the fourth research question. We could not conduct a separate meta-analysis on the type of ANT used due to the lack of relevant data. We found out that only 1 study on ANT-RPs was included in the systematic review and there were two ANT-I studies, but the results were not comparable since one of them was made on the ADHD-diagnosed group and the second on the group of risk of ADHD.

### 3.4 Subgroup analysis

We aimed to compare symptoms of ADHD at intensity A against symptoms at intensity B to check whether the severity of the symptoms of attention disorders is reflected in the results of the ANT. We assumed that children with ADHD diagnoses are a heterogeneous group with different intensities of attention deficit. Even in the DSM 5 diagnostic criteria, a child should obtain a minimum of 6 out of 9 criteria of inattention to gain an ADHD diagnosis. However, due to the lack of relevant data we could not conduct a subgroup analysis.

We planned to compare type A of ADHD associated with characteristics of inattention vs. type B of ADHD associated with characteristics of hyperactivity to check whether the results of the ANT are different for each ADHD type. Each type of ADHD is tied to one or more core symptoms. If such a difference were identified, it would be possible to predict the ADHD type based on the ANT results and to describe the pattern of the ANT results for every type of ADHD. Because the distinction between different types of ADHD was not reported in almost all studies included, we could not conduct a subgroup analysis.

### 3.5 Study quality

Results for the evaluation of study quality are presented in Table 3. The majority of the studies included in the meta-analysis were of high quality (the scores ranged from 5 to 9, with an average score of 7.89). Three studies (Forssman et al., 2009; Forns et al., 2014; Julvez et al., 2020) were assessed as poor quality due to the comparability of cohorts on the basis of the design or analysis. We judged the study quality in selection of participants for the study (representativeness of the exposed cohort) to be good for 15 studies and poor for 3 studies (Forssman et al., 2009; Forns et al., 2014; Julvez et al., 2020). Selection of the non-exposed cohort was assessed as having a low risk of bias (good quality) for 16 studies and high risk (poor quality) for two studies (Waldon et al., 2018; Abramov et al., 2019). Ascertainment of exposure was judged as having good for 13 studies and poor for 4 studies (Forssman et al., 2009; Forns et al., 2014; Antonini et al., 2016; Chen et al., 2022) implicated that in the majority of the included studies ADHD diagnosis was based on secure record according to psychiatrics’ or psychologists’ judgment (e.g., the subjects were evaluated jointly by a psychiatrist and a neuropediatric in an interview based on the DSM criteria and associated with the application of these criteria to the children’s parents), structured interview or self-repotted questioners (such the CONNERS 3 – Questionnaires for ADHD assessment). Demonstration that outcome of interest was not present at the start of the study was applied to all studies, where the ANT performance was provided during data collection period, not at the same time point as diagnosis of ADHD was made. In terms of bias due to comparability, the comparability of cohorts on the basis of the design or analysis was assessed as being good for 15 studies and poor for 3 studies (Forssman et al., 2009; Forns et al., 2014; Julvez et al., 2020). This means that selection of exposed and unexposed in cohort studies was from similar populations in relation to age and gender (which were established as the most important factor). The cohorts were not comparable for confounding factors in only three studies. In assessing potential bias due to ANT outcome misclassification, the review authors assessed all four domains (assessment of ANT outcome, length of follow-up, adequacy of follow up of cohorts) as having low risk of bias (good quality) for all 18 studies and high risk for no studies. In all studies assessment of ANT was based on the application of standard version of ANT (such as: ANT, ANT-C), follow-up was long enough for outcome to occur and the number of lost to follow up less or equal to 20% or description of those who lost suggested not different from those who were followed.

**TABLE 3:** Summary of the study quality based on the Newcastle – Ottawa quality assessment scale for cohort studies.

Domain	Abramov et al., 2019	Adólfsdóttir et al., 2008	Antonini et al., 2016	Booth et al., 2007	Chen et al., 2022	Forns et al., 2014	Forssman et al., 2009	Gupta et al., 2011	Hansen et al., 2014	Johnson et al., 2008	Julvez et al., 2020	Konrad et al., 2010	Kooistra et al., 2011	Kratz et al., 2011	Mogg et al., 2015	Mullane et al., 2011	Racicka-Pawlukiewicz et al., 2021	Waldon et al., 2018	Quality of study evalua-tion*
**Selection** Representa-tiveness of the exposed cohort	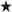	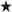	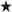	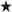	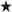			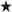	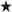	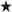		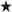	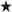	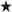	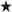	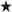	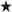	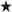	15 stars
**Selection** Selection of the non-exposed cohort		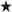	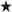	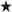	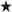	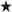	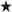	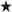	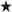	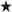	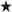	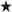	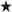	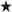	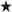	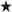	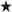		16 stars
**Selection** Ascertainment of exposure	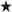	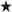		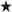				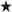	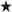	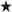		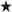	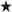	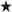	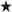	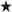	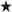	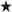	13 stars
**Selection** Demonstration that outcome of interest was not present at start of study	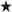	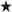	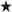	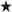	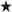	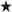	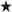	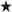	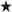	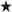	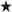	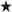	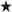	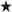	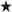	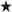	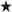	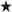	18 stars
**Comparability** Comparability of cohorts on the basis of the design or analysis	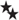	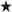	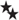	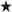	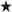			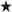	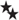	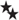		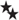	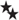	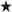	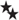	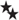	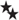	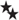	26 stars
**Outcome** Assessment of outcome	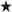	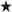	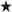	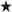	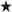	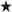	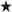	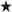	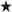	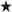	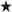	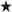	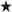	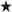	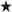	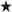	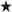	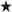	18 stars
**Outcome** Was follow-up long enough for outcomes to occur	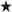	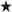	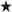	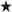	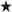	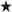	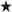	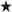	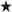	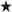	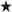	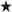	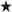	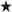	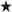	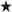	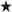	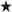	18 stars
**Outcome** Adequacy of follow up of cohorts	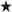	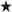	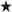	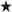	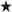	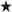	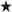	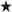	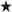	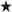	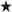	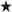	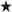	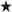	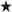	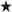	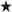	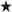	18 stars
Quality of study evaluation*	Good	Good	Good	Good	Good	Poor	Poor	Good	Good	Good	Poor	Good	Good	Good	Good	Good	Good	Good	Mean: 7.89

A study can be awarded a maximum of one star for each numbered item within the Selection and Outcome categories. A maximum of two stars can be given for Comparability.

*Good quality: 3 or 4 stars in selection domain AND 1 or 2 stars in comparability domain AND 2 or 3 stars in outcome/exposure domain. Fair quality: 2 stars in selection domain AND 1 or 2 stars in comparability domain AND 2 or 3 stars in outcome/exposure domain. Poor quality: 0 or 1 star in selection domain OR 0 stars in comparability domain OR 0 or 1 stars in outcome/exposure domain. Study IDs marked in gray were included in the main meta-analysis.

### 3.6 Certainty of evidence

Overall quality of evidence with the GRADE evaluation along with the results are presented in Table 4. Because only observational studies were included, we started the rating with “moderate” quality for all outcomes. In the end, the evidence for three outcomes (Executive Network scores; Alerting Network scores and Orienting Network Scores, all for ADHD) was judged to be *“moderate,”* while other for the two outcomes (Reaction time and Reaction time variability, both for ADHD) it was “*low.”* Executive Network scores, Alerting Network scores, Orienting Network Scores, Correctness, Reaction time and Reaction time variability (all for risk of ADHD) were of “*very low evidence.”*

**TABLE 4:** Overall quality of evidence and the GRADE evaluation.

ANT outcomes	Studies	Population			Mean diffe-rences (95% CI)	Hetero-geneity	Certainty of evidence GRADE				Quality assess-ment	Key messages in simple terms
		N of ADHD	N of at risk	N of non-ADHD			Risk of bias	Inconsis-tency	Indirect-ness	Impreci-sion		
Executive Network scores in ADHD	5 studies (Booth et al., 2007; Adólfsdóttir et al., 2008; Kooistra et al., 2011; Hansen et al., 2014; Mogg et al., 2015)	217		880	0,126 [−0.059; 0.312]	*I*^2^ = 6.92%	No serious limitations, do not downgrade	No serious limitations, do not downgrade	No serious limitations, do not downgrade	No serious limitations, do not downgrade	⊕⊕⊕⊕	Non-significant pooled effects for executive network scores between children with ADHD and controls
Executive Network scores in at risk	2 studies (Forns et al., 2014; Antonini et al., 2016)		373	2,356	0.632 [−0,116; 1.380]	*I*^2^ = 93.17%	Serious limitations, downgrade one level	Very serious limitations, downgrade two levels	Very serious limitations, downgrade two levels	Serious limitations, downgrade one level		Non-significant pooled effects for executive network scores between children at risk of ADHD and controls
Alerting Network scores in ADHD	5 studies (Booth et al., 2007; Adólfsdóttir et al., 2008; Kooistra et al., 2011; Hansen et al., 2014; Mogg et al., 2015)	217		880	0.235 [0.021; 0.449]	*I*^2^ = 27.70%	No serious limitations, do not downgrade	No serious limitations, do not downgrade	No serious limitations, do not downgrade	No serious limitations, do not downgrade	⊕⊕⊕⊕	Significant higher pooled effects for alerting network scores in children with ADHD compared to controls
Alerting Network scores in at risk	2 studies (Forns et al., 2014; Antonini et al., 2016)		373	2,356	0.176 [0.003; 0.349]	*I*^2^ = 23.67%	Serious limitations, downgrade one level	No serious limitations, do not downgrade	Very serious limitations, downgrade two levels	No serious limitations, do not downgrade	⊕⊕	Significant higher pooled effects for alerting network scores in children at risk of ADHD compared to controls
Orienting Network Scores in ADHD	5 studies (Booth et al., 2007; Adólfsdóttir et al., 2008; Kooistra et al., 2011; Hansen et al., 2014; Mogg et al., 2015)	217		880	0.109 [−0.069;0.286]	*I*^2^ = 0.00%	No serious limitations, do not downgrade	No serious limitations, do not downgrade	No serious limitations, do not downgrade	No serious limitations, do not downgrade	⊕⊕⊕⊕	Non-significant pooled effects for orienting network scores between children with ADHD and controls
Orienting Network Scores in at risk	2 studies (Forns et al., 2014; Antonini et al., 2016)		373	2,356	−0.001 [−0.120; 0.117]	*I*^2^ = 0.00%	Serious limitations, downgrade one level	No serious limitations, do not downgrade	Very serious limitations, downgrade two levels	No serious limitations, do not downgrade	⊕⊕	Non-significant pooled effects for orienting network scores between children at risk of ADHD and controls
Correctness in ADHD	3 studies (Booth et al., 2007; Adólfsdóttir et al., 2008; Mogg et al., 2015)	132		897	0.393 [−0.055; 0.841]	*I*^2^ = 73.62%	No serious limitations, do not downgrade	Serious limitations, downgrade one level	No serious limitations, do not downgrade	Serious limitations, downgrade one level	⊕⊕	Non-significant pooled effects for number of errors scores in children at high risk of ADHD compared to controls indicating they make similar number of errors.
Correctness in at risk	2 studies (Forns et al., 2014; Antonini et al., 2016)		373	2,356	1.956 [0.020; 3.892]	*I*^2^ = 98.29%	Serious limitations, downgrade one level	Very serious limitations, downgrade two levels	Very serious limitations, downgrade two levels	Serious limitations, downgrade one level		Significant higher pooled effects for number of errors scores in children at high risk of ADHD compared to controls indicating that they make more mistakes.
Reaction time in ADHD	5 studies (Booth et al., 2007; Adólfsdóttir et al., 2008; Kooistra et al., 2011; Hansen et al., 2014; Mogg et al., 2015)	217		880	0.433 [0.135;0.731]	*I*^2^ = 61.02%	No serious limitations, do not downgrade	Serious limitations, downgrade one level	No serious limitations, do not downgrade	No serious limitations, do not downgrade	⊕⊕⊕	Significant pooled effects for differences in the ratio times in a particular type of clue or flankers measured with the ANT in children with ADHD compared to controls indicating that need more time for reaction.
Reaction time in at risk	2 studies (Forns et al., 2014; Antonini et al., 2016)		373	2,356	0.593 [−0.257; 1.444]	*I*^2^ = 94.70%	Serious limitations, downgrade one level	Very serious limitations, downgrade two levels	Very serious limitations, downgrade two levels	Serious limitations, downgrade one level		Non-significant pooled effects for differences in the ratio times in a particular type of clue or flankers measured with the ANT in children at risk of ADHD compared to controls.
Reaction time variability in ADHD	2 studies (Adólfsdóttir et al., 2008; Mogg et al., 2015)	90		783	0.334 [0.012; 0.657]	*I*^2^ = 41.65%	No serious limitations, do not downgrade	No serious limitations, do not downgrade	No serious limitations, do not downgrade	Serious limitations, downgrade one level	⊕⊕⊕	Significant pooled effects for differences in the ratio times in a particular type of clue or flankers measured with the ANT in children with ADHD compared to controls meaning reaction time variability was higher.
Reaction time variability in at risk	2 studies (Forns et al., 2014; Antonini et al., 2016)		373	2,356	5.447 [−5.011; 15.904]	*I*^2^ = 99.61%	Serious limitations, downgrade one level	Very serious limitations, downgrade two levels	Very serious limitations, downgrade two levels	Serious limitations, downgrade one level		Non-significant pooled effects for differences in the ratio times variability in a particular type of clue or flankers measured with the ANT in children at risk of ADHD compared to controls.

Evidence was judged as “high,” “moderate,” “low,” or “very low” quality depending on the extent to which we could be certain that the pooled effect estimate is close to the true effect. Certainty of evidence of evidence is expressed by means of “⊕” figures (⊕⊕ low; ⊕⊕⊕ moderate; and ⊕⊕⊕⊕ high). The quality of evidence was downgraded by 1 level for each of the following reasons: (1) high risk of bias across the studies, (2) indirectness of evidence (indirect population, intervention, control, outcomes), (3) high heterogeneity (*I*^2^ > 50%) or inconsistency of results across studies, (4) imprecision of results (wide confidence intervals, small sample size). Details on grading of the certainty of the evidence are presented in Supplementary Table 3.

## 4 Discussion

Although ADHD and attention measured with the attention network test (ANT) has long been of interest (Vázquez-Marrufo et al., 2019; Arora et al., 2020); to the best of our knowledge, this is the first meta-analysis to examine patterns of ANT performance results for children with ADHD and at risk of ADHD including the efficiency of the attention network, reaction time, and number of all types of errors.

### 4.1 Executive, alerting, and orienting networks

Our primary question pertained to differences in the efficiency of functioning of the three attention networks measured with the ANT between children diagnosed with ADHD, at risk of ADHD, and children without ADHD. Based on our pooled data, we did not observe significant differences in Executive Network scores between children with ADHD and controls nor children at risk of ADHD and controls. Interestingly, there were significant pooled effects for Alerting Network scores, which were significantly higher in both children with ADHD and children at risk of ADHD compared to children without ADHD. The most surprising result is that no significant differences were found between children with ADHD or children at risk of ADHD and children without ADHD for the orienting network. Taken together, these results seem to suggest that children with ADHD and at risk of ADHD perform the ANT differently to children without ADHD only in terms of the alerting network, while executive and orienting outcomes did not manifest in any difference in behaviors. There are several possible explanations for this result.

Focusing on an attentional alerting network, one study by Hansen et al. (2014) stands out from other studies involved in the analyses (an outliner). We believe that this is due to the methodological issues such as diagnostic measurement tool used (the Schedule for Affective Disorders and Schizophrenia for School-age Children - Present and Lifetime Version) dedicated to anxiety, depression, mania, obsessive-compulsive disorder, anorexia nervosa, etc., rather than ADHD. In addition, the version of the diagnostic tool used in this study dates back to 1997 – as we know, quite a bit in the diagnosis of ADHD has moved forward since then. Third, we assume that the target population might not fully reflect a typical group of children with ADHD because of the more inclusive diagnostic criteria used for the study purpose.

The between-group differences in the efficiency of alerting indicate the specific dysfunction of this attentional network in ADHD. Rueda et al. (2004) and Posner et al. (2012) maintain that older children and adults should rely on internal tonic alertness rather than external signal warnings (typical for younger children). In younger children, they observed much higher scores for attentional alerting on the ANT-C, while the scores for older children and adults were much smaller. For instance, in the study of Rueda et al. (2004), children aged 10 years had greater scores than adults for attentional alerting on ANT-C, as they had longer response times for conditions with no alerting clues. Thus, the obtained result in our meta-analysis may reflect underdevelopment of the alerting network in children with ADHD.

Attentional alerting has a neural basis in the posterior area, thalamus, locus coeruleus, and frontal area (Posner and Rothbart, 2007; Posner and Fan, 2008). The meta-analysis of Dickstein et al. (2006) found that individuals with ADHD present with hypoactivation mostly in the fronto-striatal and parietal regions. In a study conducted by Cortese et al. (2012), children with ADHD, in comparison to controls, presented hypoactivation in the frontoparietal attention network (including the lateral frontal pole, dorsal anterior cingulate, dorsolateral anterior prefrontal cortex, lateral cerebellum, anterior insula, and inferior parietal lobe) and ventral attention network (including the temporoparietal junction, the supramarginal gyrus, frontal operculum, and anterior insula). Hyperactivation was observed predominantly in the default network, but also in the somatomotor and visual systems. This observation is consistent with the classical model of ADHD as a disorder of deficient fronto-striatal activation (Castellanos, 1997) and supports a model of ADHD based on interrelationships among neural networks (Cortese et al., 2012). Banich et al. (2009) found that young adults with ADHD performing attentional tasks present neural dysregulation across brain regions, including those involved in overall arousal, top-down attentional control, response selection, and inhibition. For sustained attentional control, the dysregulation was the greatest in lateral areas of the dorsolateral prefrontal cortex, while for transient aspects of attentional control it was greatest in medial regions of the dorsolateral prefrontal cortex. Furthermore, using a fNIRS-based method, Ikeda et al. (2022) found robustly lower hemodynamic responses in the right prefrontal cortex during an inhibition task (a go/no-go task) in children with ADHD in comparison to controls. According to Castellanos and Proal (2012) as well as Makris et al. (2009), in ADHD, we can observe disturbed connectivity within and among several neural networks, rather than abnormalities of discrete, isolated brain regions.

The obtained results have clinical implication. The significant differences in the attentional alerting in comparison with non-significant differences in executive attention and orienting point out that children with ADHD or at risk of ADHD experience particular difficulties in maintaining internal tonic alertness during prolonged cognitive activities. While the ability to orient attention to external stimuli as well as the ability to deal with distracting stimulus seems to be less affected. From the diagnostic point of view, the results stress that the assessor should be focused more on specific diagnostic criteria dedicated to inattentive such us: experience difficulties staying alert on tasks or activities (e.g., reading or writing), following instructions and completing ongoing schoolwork, losing quickly focus, avoiding assignments requiring sustained mental effort. Because these aforementioned diagnostic symptoms in the light of obtained outcomes seem to be key symptoms helping to differencing children from clinical group from non-ADHD group. It follows from this as well, intervention should be focused on resources linked with being able to maintain alertness rather than executive attention or orienting of attention.

### 4.2 Correctness

One of the secondary questions in this meta-analysis was whether primary-school-age children diagnosed with ADHD or at risk of ADHD show differences in Correctness scores compared to children without ADHD, including numbers of Commission and Omission Errors, measured with ANT. The current review found that children at risk of ADHD had higher error scores compared with controls, indicating that they make more mistakes; this was not observed in the group of children diagnosed with ADHD. A possible explanation might be that children who had already been diagnosed with ADHD participate in therapies focused on improving such abilities. There are, however, other possible explanations. Although other researchers (Klee and Garfinkel, 1983; Eliason and Richman, 1987; Barkley, 1991; Halperin et al., 1991; Corkum and Siegel, 1993; Lassiter et al., 1994; Epstein et al., 1998; Inoue et al., 1998) have indicated that omission errors reflect symptoms of inattention, Barkley (1991) found that correlations between omission errors and measures of inattention are low to moderate. Moreover, according to Losier et al. (1996), children with ADHD treated with methylphenidate present significant reductions in the rate of omission errors. Perhaps this is why such errors manifested only in the ADHD risk group in our meta-analysis, as these children participate in therapies (incl. pharmacotherapy) more rarely than diagnosed children and thus symptoms of inattention are much greater in this cohort. The obtained results have also clinical implication. From therapeutic perspective, it would be more effective if therapy would have focused on reflective and slower work approach, which would contribute to less errors made.

### 4.3 Reaction time and reaction time variability

The next secondary question was whether primary-school-aged children diagnosed with ADHD or who are at risk of ADHD demonstrate differences relative to children without ADHD in reaction times on a particular type of clue or flanker task measured with the ANT. The interesting and clinically relevant finding was that Reaction Time was longer and Reaction Time Variability higher in children with ADHD than in children without ADHD and children at risk of ADHD compared with children without ADHD. Of note, Epstein et al. (2011) as well as Karalunas et al. (2012) described these findings earlier in their studies. It seems possible that this general slowing might indicate a variety of unspecified difficulties with basic cognitive processes (Hervey et al., 2006), and higher Reaction Time Variability reflects greater fluctuations and lapses of attention among children with ADHD (Van der Molen, 1996). Several researchers (Douglas, 1999) have noted that performance variability is important in ADHD and reaction time is an important reflection of attention. The results of our meta-analysis indicate that it is not the case that children with ADHD cannot pay attention at all, but rather they present attention lapses more frequently than do controls. According to Castellanos and Tannock (2002), performance variability is the essence of ADHD and temporal and contextual variables play an important role in this inconsistency. Neuroimaging studies (Giedd et al., 2001) have identified that response variability is related to a distributed brain network including the frontal lobes, which is consistently implicated in the pathophysiology of ADHD.

All activities that require time control or are under time limit will make challenge for the school-age children due to their limitations. The obtained results have practical implication suggesting that children from clinical group (ADHD and at risk of ADHD) will need more time to follow instructions, complete ongoing tasks or experience challenges in time managing. It also refers to the observation of high prevalence of learning disorders comorbidity among children with ADHD.

### 4.4 Implications for research and practice

The findings of this review have important implications for future practice. It may be possible that the characteristic patterns of ANT results in children with ADHD can be used as a reference point to help determine whether a given intervention (psychological, pharmacological, or other) used to reduce attention deficits in children is effective and results in actual, statistically significant differences in the efficiency of the attention networks. We anticipate that our review could bring rationale for researchers to furthering research into the use of the ANT for more comprehensive diagnosis of attention disorders in children, including ADHD.

### 4.5 Limitations

Some limitations of these results must be acknowledged, both at the review level and at the study level.

First, we used a comprehensive search strategy including both searching electronic databases and manually searching the reference lists of the included studies; as such, we believe this systematic review contains all relevant studies that have been conducted in this field. However, the searching strategy was limited to MeSH heading for Attention Deficit Disorder with Hyperactivity according to DSM-V. In addition, it is possible that there are unpublished studies (e.g., conference abstracts and other gray literature) of which we are not aware. Unpublished studies can be an important source of studies for inclusion in reviews.

Second, despite an exhaustive search, we were not able to identify intervention studies; thus, only prospective cohort studies were included. Intervention studies measuring treatment effects with ANT do exist, but they failed to meet the rigorous requirements of the pre-specified eligibility criteria.

Originally, we had also planned to extract information about the intensity of ADHD symptoms and use them to check whether ANT results get worse as the severity of ADHD symptoms increases (Gradys et al., 2022). However, there were considerable discrepancies in the types of tools used to diagnose ADHD, which made such a comparison unfeasible.

Forth, we also aimed to extract data on the type of ADHD (type A vs. type B) to check whether the results of the ANT are different for each ADHD type. Despite the effort expended to obtain this data, the articles included in the meta-analysis did not provide it in all cases, preventing evaluation of whether ADHD type predicts the pattern of ANT results.

Further, we aimed to check whether the version of the ANT used affects the results obtained by all children. However, it was not possible to answer this research question because the majority of studies used the same ANT version (namely ANT-C). The fact that we could not meet all the objectives of this systematic review means that further research should be conducted, as some questions still remain unanswered.

Fifth, the limited number of studies per outcome (e.g., only two studies had data on Reaction Time Variability scores for children with ADHD and at risk of ADHD) impacted that we were not able to conduct any sub-group analysis due to the lack of relevant data.

The comprehensiveness of our review was also impacted by the limited sample size of children diagnosed with ADHD or at risk of ADHD compared to controls. Larger sample sizes would allow us to better test the study objectives.

Finally, the generalizability of these results is restricted to children of primary school age. In terms of the study-level limitations, there is a need to acknowledge the high heterogeneity of the internal validity of the individual studies (such as the varied presence of psychiatric comorbidities and wide array of medicated/non-medicated subjects for the ANT); thus, obtained data must be interpreted with caution. Of note, recently there has been a debate among researchers about the problematic use of difference scores, and the treatment of errors and reaction time as separable measures (e.g., Rodebaugh et al., 2016; Hedge et al., 2018). Researchers points that the difference scores (results obtained as a subtractions of basic outcomes such as the result of the subtraction of the RT in one test condition from the outcome from the other test condition) have very low reliability as individuals may present very large differences in overall RTs (for instance the difference between 2,000 ms in one condition and 3,000 ms in another condition for one person would be two times greater than the difference between 1,000 ms in the same one condition and 1,500 ms in the same another condition for the other person; in such a situation a person whose result of subtraction would be greater would be gratified for longer reactions overall). Moreover, they indicate a problem with poor correlation between difference scores made up of RTs when treated as indices of identical cognitive processes. Future studies should consider these caveats.

## 5 Conclusion

Notwithstanding the limitations, the findings indicate that children with ADHD and at risk of ADHD perform the ANT differently to children without ADHD, getting lower scores only in terms of the alerting network; executive and orienting outcomes did not manifest any difference in behaviors measured with the ANT. The results confirmed that children at risk of ADHD also made more errors (commission and omission) measured with the ANT compared to children without ADHD. Finally, reaction times were longer and reaction time variability higher in children with ADHD than in children without ADHD and in children at risk of ADHD compared with children without ADHD.

## Author contributions

ŁB took part in the conceptualization of the project, provided specialist knowledge in the systematic review methodology, conducted the project administration, supervised all the steps, resolved discrepancies during the assessment of the study quality stage, and took part in writing the original draft and the final manuscript. GG searched databases and manually searched the reference list of the included articles, screened titles and abstracts of the papers found, supported data extraction, conducted the project administration, and took part in writing the original draft as well as editing and reviewing the manuscript. ML took part in the conceptualization, assessment of the study and evidence quality, provided specialist knowledge of ADHD, raised funds for the publication of the manuscript, and supported editing and reviewing the manuscript. AMD designed, conducted, and interpreted the meta-analyses, and took part in editing and reviewing the manuscript. TW-K screened titles and abstracts for their eligibility for inclusion, resolved discrepancies during the assessment of the study quality stage, and took part in writing the original draft as well as editing and reviewing the manuscript. KS-W extracted data from the included studies, and assessment of the evidence quality. AŁ-M extracted data from the included studies, editing and reviewing the manuscript. KL and PA-W extracted data from the included studies. MH helped assess study quality. All authors reviewed and accepted the final manuscript.
