# Supplementary material for: Attention deficit in primary-school-age children with attention deficit hyperactivity disorder measured with the attention network test: a systematic review and meta-analysis
Source: Front Neurosci. 2023 Dec 7;17:1246490. doi: 10.3389/fnins.2023.1246490 (PMC10749351; doi:10.3389/fnins.2023.1246490)
Supplement: Supplementary file 1 [file Table_1.docx]

Table S1. Amendments from the protocol

| Item | Justification |
| --- | --- |
| Characteristics of the clinical population | Our original protocol aimed to report information on ADHD intensity evaluated by the results of the questionnaire (e.g., Conners 3); hoverer, due to the lack of data reported in the paper, we did not provide the details. |
| The ANT results | Our original protocol aimed to report information on how the training of the ANT was performed, how the instructions were presented, the person conducting the test and their interventions with the child during the test, and any other descriptive data about the ANT performance and conducting. Hoverer, due to the lack of that kind of data across studies, we did not provide the details. |
| Judge of publication bias for each outcome | We originally aimed to judge a presence of publication bias for each outcome graphically and quantitatively. However, because we have included less than 10 studies in the meta-analysis, we decided to do not construct a funnel plot and test for asymmetry using Egger’s regression test, nor generate a Doi plot, which plots study-level effect sizes against a rank-based measure of precision (z-score, where the midpoint is defined by the most precise studies and the less precise studies are scattered outward towards the tails of the plot). |
