# Supplementary material for: Attention deficit in primary-school-age children with attention deficit hyperactivity disorder measured with the attention network test: a systematic review and meta-analysis
Source: Front Neurosci. 2023 Dec 7;17:1246490. doi: 10.3389/fnins.2023.1246490 (PMC10749351; doi:10.3389/fnins.2023.1246490)
Supplement: Supplementary file 3 [file Table_3.docx]

Table S3. Grading of the certainty of the evidence.

| Domain | Upgrading | Evaluation |
| --- | --- | --- |
| Risk of bias across the studies | The quality of evidence will be downgraded in case of high risk of based on the Newcastle-Ottawa Scale. | Most information is from studies at low risk of bias = No serious limitations, do not downgrade;  Most information is from studies at low or unclear risk of bias = No serious limitations, do not downgrade  The proportion of information from studies at high risk of bias is sufficient to affect the interpretation of results = Serious limitations, downgrade one level or Very serious limitations, downgrade two levels |
| Inconsistency | The quality of evidence will be downgraded in case of high heterogeneity (*I*^2^ > 50%) or inconsistency of results across studies. | Most information is from studies at low risk of bias = No serious limitations, do not downgrade;  Most information is from studies at low or unclear risk of bias = No serious limitations, do not downgrade  The proportion of information from studies at high risk of bias is sufficient to affect the interpretation of results = Serious limitations, downgrade one level or Very serious limitations, downgrade two levels |
| Indirectness of evidence | The quality of evidence will be downgraded un case of indirect population, control, outcomes. The indirectness which becomes stronger in case of flawed measurement of both exposure and outcome as well as failure to develop and apply appropriate eligibility criteria (inclusion of control population). | Most information is from studies at low risk of bias = No serious limitations, do not downgrade;  Most information is from studies at low or unclear risk of bias = No serious limitations, do not downgrade  The proportion of information from studies at high risk of bias is sufficient to affect the interpretation of results = Serious limitations, downgrade one level or Very serious limitations, downgrade two levels |
| Imprecision of results | The quality of evidence was downgraded in case of wide confidence intervals and/or mall sample size. Since there are no clear-cut recommendations on imprecision with continuous outcomes and standardized effect measures, we were downgraded if the sample size is < 620 (calculated under standard assumptions of α = 0.05, power = 0.80, and effect size of 0.20) (Schünemann et al., 2013), and we took into consideration the width of the confidence interval around the point estimate and the range of values it includes. | Most information is from studies at low risk of bias = No serious limitations, do not downgrade;  Most information is from studies at low or unclear risk of bias = No serious limitations, do not downgrade  The proportion of information from studies at high risk of bias is sufficient to affect the interpretation of results = Serious limitations, downgrade one level or Very serious limitations, downgrade two levels |
| If there were no serious concerns about the risk of bias, we upgraded the quality of evidence by one level for a large magnitude of effect, when a dose-response gradient was observed, and/or when accounting for all plausible confounding was reduced the pooled effect or suggested a spurious effect when results show no effect (Schünemann et al., 2013). We defined a large effect according to Cohen’s convention of 0.80 (Cohen, 1992), although we recognized that this cutoff may be too strict or not equally relevant across psychological sub-disciplines (Gignac & Szodorai, 2016; Schäfer & Schwarz, 2019). In addition, to upgrade for a large effect, the lower limit of the confidence interval of the point estimate had to be at least 0.80 or greater (Schünemann et al., 2013). | | |

Cohen, J. (1992). A power primer. *Psychol Bull*, *112*(1), 155–159. <https://doi.org/10.1037//0033-2909.112.1.155>

Gignac, G. E., & Szodorai, E. T. (2016). Effect size guidelines for individual differences researchers. *Personality and Individual Differences*, *102*, 74-78. <https://doi.org/10.1016/j.paid.2016.06.069>

Schäfer, T., & Schwarz, M. A. (2019). The Meaningfulness of Effect Sizes in Psychological Research: Differences Between Sub-Disciplines and the Impact of Potential Biases [Original Research]. *Frontiers in Psychology*, *10*. <https://doi.org/10.3389/fpsyg.2019.00813>

Schünemann, H., Brożek, J., Guyatt, G., & Oxman, A. (2013). *GRADE handbook for grading quality of evidence and strength of recommendations. Updated October 2013.* . The GRADE Working Group. guidelinedevelopment.org/handbook ]
