# Supplementary material for: Attention deficit in primary-school-age children with attention deficit hyperactivity disorder measured with the attention network test: a systematic review and meta-analysis
Source: Front Neurosci. 2023 Dec 7;17:1246490. doi: 10.3389/fnins.2023.1246490 (PMC10749351; doi:10.3389/fnins.2023.1246490)
Supplement: Supplementary file 4 [file Table_4.docx]

Table S4. List of excluded studies and reasons for exclusion

|  | Reference | Reason of exclusion |
| --- | --- | --- |
| 1 | Trelles, P., Wilkinson, E., Kolevzon, A. (2018). Characterizing Attention Deficit in Children With ASD. *Journal of the American Academy of Child & Adolescent Psychiatry,* 57, S230. https://doi.org/10.1016/j.jaac.2018.09.306 | target population |
| 2 | Krone, B., Bedard, A.C., Downes, L., Downes, Q., Kirschenbaum, A., Ivanov, I., Schulz, K. (2020). Double dissociation of neuropsychological correlates for cognitive phenotypes in ADHD. *Journal of the American Academy of Child & Adolescent Psychiatry* 59, s151. https://doi.org/10.1016/j.jaac.2020.08.069 | study design |
| 3 | Luo, X., Guo, X., Zhao, Q. et al. (2022). A randomized controlled study of remote computerized cognitive, neurofeedback, and combined training in the treatment of children with attention-deficit/hyperactivity disorder. *European Child & Adolescent Psychiatry*. https://doi.org/10.1007/s00787–022-01956-1 | not able to reach full version |
| 4 | Duarte., M., Urben, S., Bader, M. (2015). Assessment of the three attentional networks in adults with Attention-Deficit/Hyperactivity Disorder. Conference paper at the 5th World Congress on ADHD “From child to Adult disorder” | target population |
| 5 | Seitz, J., Hueck, M., Dahmen, B., Schulte-Rüther, M., Legenbauer, T., Herpertz-Dahlmann, B., & Konrad, K. (2016). Attention Network Dysfunction in Bulimia Nervosa – An fMRI Study. *PloS One*, 11(1), e0161329. https://doi.org/10.1371/journal.pone.0161329 | target population |
| 6 | Filardi, M., Pizza, F., Tonetti, L., Antelmi, E., Natale, V., & Plazzi, G. (2017). Attention impairments and ADHD symptoms in adult narcoleptic patients with and without hypocretin deficiency. PloS One, 12, e0182085. https://doi.org/10.1371/journal.pone.0182085 | target population |
| 7 | Rodriguez, D. (2004). Attention networks in attention-deficit/hyperactivity disorder subtypes: An event-related potentials study. PhD Thesis. University of South Carolina. | target population |
| 8 | Isrctn (2018) Comparing psychological interventions in order to find out what treatments are effective in helping students cope and manage with challenges to their mental health. Conference paper. | study design |
| 9 | Lazarev, V.V., Pontes, M., Pontes, A.T., Vieira, J., Cunha, C.Q., Tamborino, T., et al. (2016). EEG and ERP characteris-tics of Attention Deficit Hyperactivity Disorder in children and adolescents. International journal of psychophysiology: official journal of the International Organization of Psychophysiology 108 (75). doi: 10.1016/j.ijpsycho.2016.07.243 | study design |
| 10 | Epstein, J. N., Langberg, J. M., Rosen, P. J., Graham, A., Narad, M. E., Antonini, T. N., Brinkman, W. B., Froehlich, T., Simon, J. O., & Altaye, M. (2011). Evidence for higher reaction time variability for children with ADHD on a range of cognitive tasks including reward and event rate manipulations. *Neuropsychology*, 25(4), 427–441. https://doi.org/10.1037/a0022155 | type of article (correction to an article already published) |
| 11 | Sturm, A., Ricketts, E. J., McGuire, J. F., Lerner, J., Lee, S., Loo, S. K., McGough, J. J., Chang, S., Woods, D. W., McCracken, J., & Piacentini, J. (2021). Inhibitory control in youth with Tourette's Disorder, attention-deficit/hyperactivity disorder and their combination and predictors of objective tic suppressibility. *Psychiatry Research*, 304, 114163. https://doi.org/10.1016/j.psychres.2021.114163 | ANT type |
| 12 | Bueno, V. F., Kozasa, E. H., da Silva, M. A., Alves, T. M., Louzã, M. R., & Pompéia, S. (2015). Mindfulness Meditation Improves Mood, Quality of Life, and Attention in Adults with Attention Deficit Hyperactivity Disorder. *BioMed Research International,* 2015, 962857. https://doi.org/10.1155/2015/962857 | target population |
| 13 | Stevenson, M.P., McEwan, J., Bentsen, P., Schilhab, T., Glue, P., Trani, P., Wheeler, B., Healey, D. (2021). Nature walks versus medication: A pre-registered randomized-controlled trial in children with Attention Deficit/Hyperactivity Disorder. *Journal of Environmental Psychology,* 77, 101679. https://doi.org/10.1016/j.jenvp.2021.101679 | no ANT results |
| 14 | Nattel, S., Wilkinson, E., Kristin, M.,  Isenstein, E., Kolevzon, A., Trelles, M.P. (2018). Parsing Attention Dysfunction in Children With Autism Spectrum Disorder and Attention Deficit/Hyperactivity Disorder. *Biological Psychiatry*, 83, s153 https://doi.org/10.1016/j.biopsych.2018.02.399 | target population |
| 15 | Luckhardt, C., Schütz, M., Mühlherr, A. et al. (2021). Phase-IIa randomized, double-blind, sham-controlled, parallel group trial on anodal transcranial direct current stimulation (tDCS) over the left and right tempo-parietal junction in autism spectrum disorder—StimAT: study protocol for a clinical trial. *Trials,* 22, 248. https://doi.org/10.1186/s13063-021–05172-1 | type of article  (study protocol) |
| 16 | Lo, H. H., Wong, S. Y., Wong, J. Y., Wong, S. W., & Yeung, J. W. (2016). The effect of a family-based mindfulness intervention on children with attention deficit and hyperactivity symptoms and their parents: design and rationale for a randomized, controlled clinical trial (Study protocol). *BMC Psychiatry*, 16, 65. https://doi.org/10.1186/s12888-016-0773-1 | type of article  (study protocol) |
| 17 | Forns, J., Esnaola, M., López-Vicente, M., Suades-González, E., Alvarez-Pedrerol, M., Julvez, J., Grellier, J., Sebastián-Gallés, N., & Sunyer, J. (2014). The n-back test and the attentional network task as measures of child neuropsychological development in epidemiological studies. *Neuropsychology*, 28(4), 519–529. https://doi.org/10.1037/neu0000085 | type of article  (correction to an article already published) |
| 18 | Poynter, W., Ingram, P., & Minor, S. (2010). Visual field asymmetries in attention vary with self-reported attention deficits. *Brain and Cognition*, 72, 355–361. https://doi.org/10.1016/j.bandc.2009.10.014 | target population |
