# Supplementary material for: Attention deficit in primary-school-age children with attention deficit hyperactivity disorder measured with the attention network test: a systematic review and meta-analysis
Source: Front Neurosci. 2023 Dec 7;17:1246490. doi: 10.3389/fnins.2023.1246490 (PMC10749351; doi:10.3389/fnins.2023.1246490)
Supplement: Supplementary file 5 [file Table_5.docx]

Table S5. List of duplicates studies and reasons for merging

|  | Reference | Reason of merging | Item included in a systematic review |
| --- | --- | --- | --- |
| 1 | Suades-González, E., Forns, J., García-Esteban, R., López-Vicente, M., Esnaola, M., Álvarez-Pedrerol, M., Julvez, J., Cáceres, A., Basagaña, X., López-Sala, A., & Sunyer, J. (2017). A Longitudinal Study on Attention Development in Primary School Children with and without Teacher-Reported Symptoms of ADHD. *Frontiers in Psychology*, 8, 655. https://doi.org/10.3389/fpsyg.2017.00655 | the same project: BREATH Project | Forns et al. (2014). The n-back test and the attentional network task as measures of child neuropsychological development in epidemiological studies. *Neuropsychology,* 28(4), 519-529. https://doi.org/10.1037/neu0000085 |
| 2 | Forns, J., Esnaola, M., López-Vicente, M., Suades-González, E., Alvarez-Pedrerol, M., Julvez, J., Grellier, J., Sebastián-Gallés, N., & Sunyer, J. (2014). The n-back test and the attentional network task as measures of child neuropsychological development in epidemiological studies. *Neuropsychology*, 28(4), 519–529. https://doi.org/10.1037/neu0000085 |  |  |
| 3 | Camprodon-Rosanas, E., Ribas-Fitó, N., Batlle, S., Persavento, C., Alvarez-Pedrerol, M., Sunyer, J., & Forns, J. (2020). Association Between Sluggish Cognitive Tempo Symptoms and Attentional Network and Working Memory in Primary Schoolchildren. *Journal of Attention Disorders,* 24(13), 1787–1795. https://doi.org/10.1177/1087054717702245 |  |  |
| 4 | Aguilar-Lacasaña, S., Vilor-Tejedor, N., Jansen, P. R., López-Vicente, M., Bustamante, M., Burgaleta, M., Sunyer, J., & Alemany, S. (2022). Polygenic risk for ADHD and ASD and their relation with cognitive measures in school children. *Psychological Medicine*, 52, 1356–1364. https://doi.org/10.1017/S0033291720003189 |  |  |
| 5 | Neuropsychological assessment in the BREATHE project: preliminary results September 2013ISEE Conference Abstracts 2013:4013 DOI: 10.1289/isee.2013.P-3-30-12 |  |  |
| 6 | Abramov, D. M., Lazarev, V. V., Gomes Junior, S. C., Mourao-Junior, C. A., Castro-Pontes, M., Cunha, C. Q., deAzevedo, L. C., & Vigneau, E. (2019). Estimating biological accuracy of DSM for attention deficit/hyperactivity disorder based on multivariate analysis for small samples. *PeerJ*, 7, e7074. https://doi.org/10.7717/peerj.7074 | the same project | Abramov et al. (2019). Neurophysiological and behavioral correlates of alertness impairment and compensatory processes in ADHD evidenced by the Attention Network Test. *PLoS One*, 14(7), e0219472. https://doi.org/10.1371/journal.pone.0219472 |
| 7 | Abramov, D. M., Cunha, C. Q., Galhanone, P. R., Alvim, R. J., de Oliveira, A. M., & Lazarev, V. V. (2019). Neurophysiological and behavioral correlates of alertness impairment and compensatory processes in ADHD evidenced by the Attention Network Test. *PloS One*, 14, e0219472. https://doi.org/10.1371/journal.pone.0219472 |  |  |
| 8 | Kratz, O., Studer, P., Malcherek, S., Erbe, K., Moll, G. H., & Heinrich, H. (2011). Attentional processes in children with ADHD: an event-related potential study using the attention network test. *International Journal of Psychophysiology: official journal of the International Organization of Psychophysiology*, 81, 82–90. https://doi.org/10.1016/j.ijpsycho.2011.05.008 | the same project: Attenctional process |  |
| 9 | Heinrich, H., Busch, K., Studer, P., Erbe, K., Moll, G. H., & Kratz, O. (2014). EEG spectral analysis of attention in ADHD: implications for neurofeedback training? *Frontiers in Human Neuroscience*, 8, 611. https://doi.org/10.3389/fnhum.2014.00611 |  |  |
| 10 | Carrizosa, C., Murcia, M., Ballesteros, V., Costa, O., Manzano-Salgado, C. B., Ibarluzea, J., Iñiguez, C., Casas, M., Andiarena, A., Llop, S., Lertxundi, A., Schettgen, T., Sunyer, J., Ballester, F., Vrijheid, M., & Lopez-Espinosa, M. J. (2021). Prenatal perfluoroalkyl substance exposure and neuropsychological development throughout childhood: The INMA Project. *Journal of Hazardous Materials*, 416, 125185. https://doi.org/10.1016/j.jhazmat.2021.125185 | the same project: IMMA | Julvez, J. et al. (2020). Maternal seafood consumption during pregnancy and child attention outcomes: a cohort study with gene effect modification by PUFA-related genes. *International Journal of Epidemiology*, 49(2), 559-571. https://doi.org/10.1093/ije/dyz197 |
| 11 | Lozano, M., Murcia, M., Soler-Blasco, R., González, L., Iriarte, G., Rebagliato, M., Lopez-Espinosa, M. J., Esplugues, A., Ballester, F., & Llop, S. (2021). Exposure to mercury among 9-year-old children and neurobehavioural function. *Environment International*, 146, 106173. https://doi.org/10.1016/j.envint.2020.106173 |  |  |
| 12 | Julvez, J., Fernández-Barrés, S., Gignac, F., López-Vicente, M., Bustamante, M., Garcia-Esteban, R., Vioque, J., Llop, S., Ballester, F., Fernández-Somoano, A., Tardón, A., Vrijheid, M., Tonne, C., Ibarluzea, J., Irazabal, A., Sebastian-Galles, N., Burgaleta, M., Romaguera, D., & Sunyer, J. (2020). Maternal seafood consumption during pregnancy and child attention outcomes: a cohort study with gene effect modification by PUFA-related genes. *International Journal of Epidemiology*, 49, 559–571. https://doi.org/10.1093/ije/dyz197 |  |  |
| 13 | Barreto-Zarza, F., Sánchez de Miguel, M., Arranz-Freijo, E.B. et al. Family Context and ADHD Symptoms in Middle Childhood: an Explanatory Model. *Journal of Child and Family Studies*, 31, 854–865 (2022). https://doi.org/10.1007/s10826-021–02206-9 |  |  |
