# Supplementary material for: Attention deficit in primary-school-age children with attention deficit hyperactivity disorder measured with the attention network test: a systematic review and meta-analysis
Source: Front Neurosci. 2023 Dec 7;17:1246490. doi: 10.3389/fnins.2023.1246490 (PMC10749351; doi:10.3389/fnins.2023.1246490)
Supplement: Supplementary file 6 [file Table_6.docx]

S6. List of studies included in the systematic review.

1. Abramov, D. M., Cunha, C. Q., Galhanone, P. R., Alvim, R. J., de Oliveira, A. M., & Lazarev, V. V. (2019). Neurophysiological and behavioral correlates of alertness impairment and compensatory processes in ADHD evidenced by the Attention Network Test. *PLoS One*, *14*(7), e0219472. <https://doi.org/https://doi.org/10.1371/journal.pone.0219472>
2. Adólfsdóttir, S., Sørensen, L., & Lundervold, A. J. (2008). The attention network test: a characteristic pattern of deficits in children with ADHD. *Behavioral and Brain Functions*, *4*, 9. [https://doi.org/10.1186/1744-9081–4-9](https://doi.org/10.1186/1744-9081-4-9)
3. Antonini, T. N., Kingery, K. M., Narad, M. E., Langberg, J. M., Tamm, L., & Epstein, J. N. (2016). Neurocognitive and behavioral predictors of math performance in children with and without ADHD. *Journal of Attention Disorders, 20*(2), 108-118. <https://doi.org/10.1177/1087054713504620>
4. Booth, J. E., Carlson, C. L., & Tucker, D. M. (2007). Performance on a neurocognitive measure of alerting differentiates ADHD combined and inattentive subtypes: a preliminary report. *Archives of Clinical Neuropsychology*, *22*(4), 423-432. <https://doi.org/10.1016/j.acn.2007.01.017>
5. Chen, C., Li, Z., Liu, X., Pan, Y., & Wu, T. (2022). Cognitive Control Deficits in Children With Subthreshold Attention-Deficit/Hyperactivity Disorder [Original Research]. *Frontiers in Human Neuroscience*, *16*. <https://doi.org/10.3389/fnhum.2022.835544>
6. Forns, J., Esnaola, M., López-Vicente, M., Suades-González, E., Alvarez-Pedrerol, M., Julvez, J., Grellier, J., Sebastián-Gallés, N., & Sunyer, J. (2014). The n-back test and the attentional network task as measures of child neuropsychological development in epidemiological studies. *Neuropsychology*, *28*(4), 519-529. <https://doi.org/10.1037/neu0000085>
7. Forssman, L., Bohlin, G., Lundervold, A. J., Taanila, A., Heiervang, E., Loo, S., Järvelin, M.-R., Smalley, S., Moilanen, I., & Rodriguez, A. (2009). Independent Contributions of Cognitive Functioning and Social Risk Factors to Symptoms of ADHD in Two Nordic Populations-Based Cohorts. *Developmental Neuropsychology*, *34*(6), 721–735. <https://doi.org/10.1080/87565640903265111>
8. Gupta, R., Kar, B. R., & Srinivasan, N. (2011). Cognitive-motivational deficits in ADHD: development of a classification system. *Child Neuropsychology*, *17*(1), 67–81. <https://doi.org/10.1080/09297049.2010.524152>
9. Hansen, B. H., Skirbekk, B., Oerbeck, B., Wentzel-Larsen, T., & Kristensen, H. (2014). Associations between sleep problems and attentional and behavioral functioning in children with anxiety disorders and ADHD. *Behavioral Sleep Medicine*, *12*(1), 53-68. <https://doi.org/10.1080/15402002.2013.764525>
10. Johnson, K. A., Robertson, I. H., Barry, E., Mulligan, A., Dáibhis, A., Daly, M., Watchorn, A., Gill, M., & Bellgrove, M. A. (2008). Impaired conflict resolution and alerting in children with ADHD: evidence from the Attention Network Task (ANT). *Journal of Child Psychology and Psychiatry and Allied Disciplines*, *49*(12), 1339-1347. <https://doi.org/10.1111/j.1469-7610.2008.01936.x>
11. Julvez, J., Fernández-Barrés, S., Gignac, F., López-Vicente, M., Bustamante, M., Garcia-Esteban, R., Vioque, J., Llop, S., Ballester, F., Fernández-Somoano, A., Tardón, A., Vrijheid, M., Tonne, C., Ibarluzea, J., Irazabal, A., Sebastian-Galles, N., Burgaleta, M., Romaguera, D., & Sunyer, J. (2020). Maternal seafood consumption during pregnancy and child attention outcomes: a cohort study with gene effect modification by PUFA-related genes. *International Journal of Epidemiology*, *49*(2), 559-571. <https://doi.org/10.1093/ije/dyz197>
12. Konrad, K., Dempfle, A., Friedel, S., Heiser, P., Holtkamp, K., Walitza, S., Sauer, S., Warnke, A., Remschmidt, H., Gilsbach, S., Schäfer, H., Hinney, A., Hebebrand, J., & Herpertz-Dahlmann, B. (2010). Familiality and molecular genetics of attention networks in ADHD. *American Journal of Medical Genetics Part B: Neuropsychiatric Genetics*, *153B*(1), 148-158. <https://doi.org/10.1002/ajmg.b.30967>
13. Kooistra, L., Crawford, S., Gibbard, B., Kaplan, B. J., & Fan, J. (2011). Comparing Attentional Networks in fetal alcohol spectrum disorder and the inattentive and combined subtypes of attention deficit hyperactivity disorder. *Developmental Neuropsychology*, *36*(5), 566-577. <https://doi.org/10.1080/87565641.2010.549978>
14. Kratz, O., Studer, P., Malcherek, S., Erbe, K., Moll, G. H., & Heinrich, H. (2011). Attentional processes in children with ADHD: An event-related potential study using the attention network test. *International Journal of Psychophysiology*, *81*(2), 82-90. <https://doi.org/10.1016/j.ijpsycho.2011.05.008>
15. Mogg, K., Salum, G. A., Bradley, B. P., Gadelha, A., Pan, P., Alvarenga, P., Rohde, L. A., Pine, D. S., & Manfro, G. G. (2015). Attention network functioning in children with anxiety disorders, attention-deficit/hyperactivity disorder and non-clinical anxiety. *Psychological Medicine*, *45*(12), 2633-2646. <https://doi.org/10.1017/s0033291715000586>
16. Mullane, J. C., Corkum, P. V., Klein, R. M., McLaughlin, E. N., & Lawrence, M. A. (2011). Alerting, Orienting, and Executive Attention in Children With ADHD. *Journal of Attention Disorders*, *15*(4), 310-320. <https://doi.org/10.1177/1087054710366384>
17. Racicka-Pawlukiewicz, E., Kuć, K., Bielecki, M., Hanć, T., Cybulska-Klosowicz, A., & Bryńska, A. (2021). The Association between Executive Functions and Body Weight/BMI in Children and Adolescents with ADHD. *Brain Sciences*, *11*(2). <https://doi.org/10.3390/brainsci11020178>
18. Waldon, J., Vriend, J., Davidson, F., & Corkum, P. (2018). Sleep and Attention in Children With ADHD and Typically Developing Peers. *Journal of Attention Disorders*, *22*(10), 933-941. <https://doi.org/10.1177/1087054715575064>

**Conflict of Interest Statement:** The authors have declared that no competing interests exist.

**Funding information:** The "NeuroSmog: Determining the impact of air pollution on the developing brain” project is carried out within the TEAM-NET program of the Foundation for Polish Science co-financed by the European Union under the European Regional Development Fund (Nr. POIR.04.04.00-1763). The funding body does not influence the design of the study and the writing of the manuscript.

**Preregistration:** A protocol has been registered with the International Prospective Register of Systematic Reviews (PROSPERO) database (registration number: CRD42021249768).
